# Mechanisms of action of *Coxiella burnetii* effectors inferred from host-pathogen protein interactions

**DOI:** 10.1371/journal.pone.0188071

**Published:** 2017-11-27

**Authors:** Anders Wallqvist, Hao Wang, Nela Zavaljevski, Vesna Memišević, Keehwan Kwon, Rembert Pieper, Seesandra V. Rajagopala, Jaques Reifman

**Affiliations:** 1 Department of Defense Biotechnology High Performance Computing Software Applications Institute, Telemedicine and Advanced Technology Research Center, U.S. Army Medical Research and Materiel Command, Fort Detrick, Maryland, United States of America; 2 J. Craig Venter Institute, Rockville, Maryland, United States of America; Purdue University, UNITED STATES

## Abstract

*Coxiella burnetii* is an obligate Gram-negative intracellular pathogen and the etiological agent of Q fever. Successful infection requires a functional Type IV secretion system, which translocates more than 100 effector proteins into the host cytosol to establish the infection, restructure the intracellular host environment, and create a parasitophorous vacuole where the replicating bacteria reside. We used yeast two-hybrid (Y2H) screening of 33 selected *C*. *burnetii* effectors against whole genome human and murine proteome libraries to generate a map of potential host-pathogen protein-protein interactions (PPIs). We detected 273 unique interactions between 20 pathogen and 247 human proteins, and 157 between 17 pathogen and 137 murine proteins. We used orthology to combine the data and create a single host-pathogen interaction network containing 415 unique interactions between 25 *C*. *burnetii* and 363 human proteins. We further performed complementary pairwise Y2H testing of 43 out of 91 *C*. *burnetii-*human interactions involving five pathogen proteins. We used the combined data to *1*) perform enrichment analyses of target host cellular processes and pathways, *2*) examine effectors with known infection phenotypes, and *3*) infer potential mechanisms of action for four effectors with uncharacterized functions. The host-pathogen interaction profiles supported known *Coxiella* phenotypes, such as adapting cell morphology through cytoskeletal re-arrangements, protein processing and trafficking, organelle generation, cholesterol processing, innate immune modulation, and interactions with the ubiquitin and proteasome pathways. The generated dataset of PPIs—the largest collection of unbiased *Coxiella* host-pathogen interactions to date—represents a rich source of information with respect to secreted pathogen effector proteins and their interactions with human host proteins.

## Introduction

*Coxiella burnetii* is a Gram-negative intracellular bacterium, classified by the Centers for Disease Control and Prevention as a Category B biothreat agent [1–4]. It is the causative agent of Q fever, where “Q” is short for “Query” and refers to its initially unknown etiological origin. The bacterium is widespread in the United States (U.S.) and highly infectious [5,6], causing disease manifestations that range from asymptomatic, acute, and chronic, and have the ability to induce long-term sequelae [7]. Most naturally occurring infections are transmitted to humans from infected livestock, resulting in occasional localized outbreaks [8] that represent a threat to military personnel deployed to areas of endemic infection [9]. The infection is treatable with antibiotics. However, its high infection rate makes it a potential bio-warfare agent because no U.S.-licensed vaccine or specific prophylactic treatment is available. *C*. *burnetii* belongs to the phylum Proteobacteria, which contains a number of highly pathogenic intracellular bacteria. Although it shares some features with these species, it creates a uniquely adapted intracellular environment in the host cell. *C*. *burnetii* primarily infects human alveolar macrophages [10, 11], although it can infect other cell types as well [12].

The infecting bacteria use a series of mechanisms to establish themselves in the host cell, of which the most characteristic is the formation of a *Coxiella*-containing vacuole (CCV) capable of occupying the bulk of the host cell [13]. The CCV environment is unique in that it retains the characteristically low *p*H of a lysosome, an apparently essential component for optimal *C*. *burnetii* growth and metabolism. The ability to modulate the host response and cellular environment is a key adaptive aspect of this intracellular lifestyle. One essential component of this process is the secretion of bacterial proteins though the CCV membrane into the cytosol of the host cell. Although *C*. *burnetii* has components of Type I and Type II secretion systems [14,15], the most important secretory pathway is through the Dot/Icm (defect in organelle trafficking/intracellular multiplication) Type IV Secretion System (T4SS), which is known to mediate the translocation of more than 100 bacterial “effector” proteins [2,16]. The functions of these effectors include enzymatic activity and regulation of host processes, such as apoptosis [17], autophagy [18, 19], immune responses [20], and vesicular/protein trafficking [21, 22], through host-pathogen protein-protein interactions (PPIs). Less well understood is the exact nature of how these effector proteins—either as individual proteins, protein complexes, or sets of proteins, or in combination with host proteins—exert their function to establish the infection.

We have previously used pairwise host-pathogen protein interactions between a select set of putative effector proteins and whole genome human/murine protein libraries, as determined through yeast two-hybrid (Y2H) studies, to both identify virulence factors and map out the role of host-pathogen interactions in *Burkholderia mallei* and *Francisella tularensis* [23–27]. Here, we examined a select set of 33 secreted *C*. *burnetii* proteins to identify the putative host protein targets with which they interact, and used them to shed light on their possible roles in establishing and maintaining *Coxiella* infection.

The interaction data identified targeted host pathways involved in diverse sets of cellular functions, such as protein processing in the endoplasmic reticulum (ER), the innate immune response, and vacuole or organelle trafficking. We also linked individual effector-host interactions to host proteins involved in specific cellular tasks, such as cholesterol processing and cell cycle propagation at the centromere. The bulk of the interaction data was compatible with the notion that the bacteria interfere with host cell physiology at multiple intervention points, broadly corresponding to the known cellular phenotypes associated with the intracellular life-style of *Coxiella*.

## Materials and methods

### Selection of *C*. *burnetii* effector proteins for screening

We searched the literature to select *C*. *burnetii* effectors for Y2H screening according to the following criteria: *1*) the presence of the gene in the pathogenic strains Nine Mile I RSA493, Heinzerling RSA331, Dugway 5J108-111, G Q212, and K Q154; *2*) evidence that the gene is controlled by the PmrA-regulated *C*. *burnetii* T4SS stress response [28]; and *3*) evidence that the protein is secreted [16,29–31]. We obtained all *Coxiella* genomic information from the Pathosystems Resource Integration Center database [32].

### High-throughput Y2H screens to identify host-*C*. *burnetii* PPIs

#### Cloning of *C*. *burnetii* effector genes

We first cloned the *C*. *burnetii* genes into Gateway entry clones, and then the Y2H expression vectors to perform high-throughput Y2H screening against the human and mouse proteomes. To amplify the *C*. *burnetii* genes by PCR, we used gene-specific primers that incorporated forward and reverse Gateway recombination cloning sequences attB1 (5’-GGGGACAAGTTTGTACAAAAAAGCAGGCTTC-3’) and attB2 (5’-GGGGACCACTTTGTACAAGAAAGCTGGGTC-3’), respectively. We used the genomic DNA of *C*. *burnetii* RSA331 to amplify the target genes. The PCR-amplified open reading frames (ORFs) were subsequently cloned into a gateway entry vector (pDONR/zeo™), as recommended by the BP Clonase™ II enzyme provider (Thermo Fisher Scientific, Waltham, MA). We used Sanger sequencing to validate the cloned ORFs in the entry vectors. We sub-cloned the ORFs from the entry vector into yeast Y2H DNA-binding domain vectors (bait clones), pGBGT7g (as N-terminal fusion) and pGBACg (C-terminal fusion) [33], using Gateway LR reactions (Thermo Fisher Scientific). Subsequently, we transferred the Y2H bait clones into the haploid yeast strain AH109 (MAT-α), as previously described [34].

#### Auto-activation test

Before Y2H library screening, we examined the *C*. *burnetii* Y2H bait clones for auto-activation, i.e., detectable bait-dependent reporter gene activation in the absence of any interacting protein. Because the yeast strains used in this study contained the HIS3 reporter gene, auto-activation could be titrated by varying the concentration of 3-amino-1,2,4-triazole (3-AT), a competitive inhibitor of HIS3. We inspected the *C*. *burnetii* bait clones for auto-activation on synthetic yeast medium plates containing different concentrations of 3-AT. We used the lowest concentration of 3-AT that suppressed growth for auto-activation of the bait because it avoided background growth while still detecting true interactions.

#### Y2H library screening

We used a haploid yeast strain expressing each *C*. *burnetii* protein as bait for the interaction screening with human and murine normalized universal cDNA libraries (catalog nos. 630480 and 630482, respectively; Clontech Laboratories, Mountain View, CA). The bait and prey yeast culture was grown and mixed at a 1:1 ratio and plated on yeast extract, peptone, dextrose, and adenine (YEPDA) agar plates. We incubated the YEPDA agar plates at 30°C for 6 h or overnight at room temperature. During this process, both prey and bait plasmids were combined in diploid yeast cells by yeast mating. Yeast cells from the mating plates (YEPDA agar) were collected and transferred onto interaction-selection plates with yeast-synthetic medium (lacking tryptophan, leucine, and histidine) containing predefined concentrations of 3-AT, and the plates were incubated at 30°C for 4 to 6 days. We identified samples that showed colony growth on the interaction-selection plates but not on the control plates (bait mated to empty prey vector) as two-hybrid positive yeast clones. We manually selected positive yeast colonies and subjected them to yeast colony PCR followed by DNA sequencing to determine the interacting proteins [34]. We performed the Y2H screens twice for each effector and combined the data from both screens. However, we discarded single hits, i.e., interactions based on only one positive yeast colony.

All bait proteins were mapped to their corresponding *C*. *burnetii* locus tags, and all prey proteins were mapped to their official gene symbols as defined in the HUGO Gene Nomenclature Committee database [35] or Mouse Genome Informatics database [36]. When identified prey proteins could not be mapped to protein-coding sequences, we removed the interactions involving them. Moreover, we removed protein interactions between *C*. *burnetii* and “sticky” host proteins known to be indiscriminate binders as listed in S1 Table.

### Complementary pairwise Y2H testing of human-*C*. *burnetii* high-throughput protein interactions

To test *C*. *burnetii*–human protein interactions identified by the Y2H library screening, we selected 94 pairs (involving 82 human proteins) for pairwise Y2H assay testing. We randomly selected these effector-host interactions among four relatively uncharacterized *C*. *burnetii* effector proteins (CBU0794, CBU0881, CBU1724, and CBU2078) and the plasmid protein CBUA0014 based on their large number of observed interactions in the high-throughput screens. We constructed the human prey Y2H clones by sub-cloning the ORFs from the Human ORFeome collection [37] into pGADT7g and pGADCg Y2H prey vectors. We successfully cloned 79 of the 82 human ORFs into Y2H prey vectors. We transferred the Y2H prey clones into yeast strain Y187 (MAT-a), and tested 81 of the 94 selected protein interactions, using the same procedure as outlined above to identify two-hybrid positive interactions in this screen.

### Creation of expanded human-*C*. *burnetii* protein interaction network

We extracted human-murine orthologs from the NCBI HomoloGene database of homologs (www.ncbi.nlm.nih.gov/homologene) [38] and used them to identify orthology-based human-*C*. *burnetii* protein interactions [24]. We added the predicted orthology-based protein interactions to the human-*C*. *burnetii* Y2H data to create an expanded set of *C*. *burnetii*-human protein interactions. We used both human and murine libraries to provide better coverage of interactions with pooling of data allowing us to do a more robust statistical analysis. The data at hand do not support a statistical analysis of either species alone as meaningful. The provided data in the Supplementary Materials provide the species-distinct human and murine interactions.

### Gene Ontology and pathway enrichment analysis of host genes

We performed standard enrichment analyses for *C*. *burnetii*-interacting host proteins as described previously [26]. Briefly, the enrichment of Gene Ontology (GO) [39] and Kyoto Encyclopedia of Genes and Genomes (KEGG) [40] pathways was calculated in R by using the Bioconductor packages BioMart [41] and KEGGgraph [42]. The background set of proteins for the GO analysis involved all constituent proteins from the human PPI network, and we used the complete GO tree annotation, excluding the root and the top two levels of GO terms. The background set of proteins for the KEGG enrichment analysis involved human proteins available in KEGGgraph that participated in at least one KEGG pathway. We used the Benjamini-Hochberg method [43] to correct all obtained p-values (*p*_raw_) to adjusted p-values (*p*_adj_).

## Results

### High-throughput Y2H screening of host-pathogen interactions

We successfully cloned and prepared 33 *C*. *burnetii* effectors, and tested them in Y2H assays against both human and murine whole proteome libraries. Table 1 summarizes the available effector information, number of interacting host-pathogen proteins of each effector protein with either human or murine proteins, and protein interactions common to the two libraries. Of the tested pathogen proteins, 25 (76%) tested positive for interactions with either human or murine proteins, 12 (36%) showed interactions with both hosts, and eight failed to show any positive hits in our screens. The protein interaction data consisted of 273 unique interactions between 20 *C*. *burnetii* and 247 human proteins, and 157 between 17 *C*. *burnetii* and 137 murine proteins. The majority of *C*. *burnetii* proteins interacted with unique host proteins, i.e., 228 (92%) human proteins and 123 (90%) murine proteins interacted with a single *C*. *burnetii* protein. *C*. *burnetii* proteins that interacted with multiple proteins from either host also tended to interact with the other host. Of the nine pathogen effectors with more than 10 interacting host proteins, seven interacted with both hosts (7/9 or 78%). Of the remaining 16 that interacted with 10 or fewer host proteins, only five interacted with both hosts (5/16 or 31%). We observed far fewer instances of individual host-pathogen PPIs in both libraries; the Y2H screens identified five conserved PPIs between the human and murine data sets, i.e., interactions in which human proteins interacted with the same *C*. *burnetii* proteins as their murine orthologs. These differences could be due to low quantities of a prey gene in one of the two cDNA libraries or to non-exhaustive sampling of host-prey and pathogen-bait protein interactions.

**Table 1:** List of proteins evaluated in high-throughput yeast two-hybrid assay and number of host protein-protein interactions.

**Locus ID**	**Name**	**Description and Notes**	**PmrA/ Secreted**	**Protein-protein interactions**		
				Human	Murine	Shared
CBU0041	coxCC1, cirA	-	y/-	3	6	-
CBU0077	-	Hypothetical membrane spanning protein; late expression	y/y	3	3	-
CBU0175	coxK1	Ser/Thr protein kinase protein	-/y	9	-	-
CBU0295	-	Uncharacterized	-/y	1	3	-
CBU0388	cetCb2	Uncharacterized	-/y	1	-	-
CBU0410	coxCC3	Hypothetical membrane spanning protein	y/y	-	-	-
CBU0425	cirB	Uncharacterized; no intracellular replication defect	y/-	-	-	-
CBU0447	ankF	Ankyrin repeat protein; requires chaperone icmS	-/y	1	-	-
CBU0626	cetCb3	Uncharacterized	-/y	-	-	-
CBU0781	ankG	Putative ankyrin repeat protein; confirmed anti-apoptotic, requires chaperone icmS	-/-	33	7	-
CBU0794	coxCC4	Uncharacterized; trafficking to host-cell nucleus	-/y	17	5	-
CBU0881	coxCC5	Hypothetical cytosolic protein; RSA493, Q212, and Hentzerling only	-/y	59	16	-
CBU0885	-	Hypothetical cytosolic protein	-/y	2	-	-
CBU0937	coxDFB1, cirC	UPF0422 protein; no intracellular replication defect	-/y	26	-	-
CBU1217	coxU2	Hypothetical membrane spanning protein	-/y	-	-	-
CBU1314	coxCC6	Hypothetical cytosolic protein; trafficking to host-cell nucleus	-/y	3	2	-
CBU1379a	coxK2	Uncharacterized	-/y	7	-	-
CBU1425	coxDFb4	17 kDa common-antigen; surface antigen	-/y	-	-	-
CBU1457	coxTPR1	Tetratricopeptide repeat family protein	-/y	9	24	-
CBU1460	coxCC7, cig44	Uncharacterized	y/y	-	5	-
CBU1524	caeA	Anti-apoptotic	-/-	35	-	-
CBU1543	coxCC10, cig49	Uncharacterized	y/y	-	1	-
CBU1556	coxCC11, cvpC	Hypothetical membrane spanning protein; no intracellular replication defect	-/y	1	3	-
CBU1569	coxCC12	Hypothetical cytosolic protein; no intracellular replication defect	-/y	-	-	-
CBU1686	cetCb5	Uncharacterized	-/y	-	-	-
CBU1724	cetCb6	Uncharacterized	-/y	17	37	2
CBU1751	coxDFB5, cig57	Vesicular trafficking	y/y	-	2	-
CBU1769	coxH3	Alpha/beta hydrolase	-/y	5	-	-
CBU1823	coxH4, cig61, icaA	Uncharacterized	y/y	-	-	-
CBU1825	coxDFB6	Uncharacterized	-/y	-	5	-
CBU2056	-	Uncharacterized	-/y	-	4	-
CBU2078	coxFIC1	Fic family protein	-/y	15	27	1
CBUA0014	coxU3	Uncharacterized; Hentzerling and RSA493 only	y/y	26	7	1

The binary interactions for all human and orthologous murine proteins are detailed in S2 Table.

Although Weber et al. reported that CBU0041 and CBU0885 were toxic to yeast when their expression was strongly induced in yeast [16], these proteins were not toxic in our Y2H screening using *Saccharomyces cerevisiae* AH109 and Y187 strains. The yeast Y2H vectors we used have a “2-micron” (2μ) origin where an endogenous (ADH1) promoter–resulting in a low-level expression of the recombinant protein–drives the protein expression and, hence, resulting in a lack of toxicity in our experiments.

The Y2H screens failed to capture the previously identified interaction between AnkG (CBU0881) and p32 [17]. A Y2H failure to detect (false negative) may have multiple origins, such as lack of exhaustive screening, true absence under the current set of experimental conditions, or deficiency of the target protein in the library (p32 was not detected in any other interaction in our screens). Currently, we are not able to determine the definitive cause of this absence.

In the following sections, we used the generated data to broadly characterize possible host-pathogen interaction phenotypes by performing overall analyses that take into account sets of observed and pooled interactions. These analyses do not allow us to individually account for each protein interaction, because individual protein-protein interactions range from strong binding events to more ephemeral signaling events and, as such, are not fully characterized or distinguishable by the deployed Y2H technique.

### Orthology-derived high-throughput human-*C*. *burnetii* protein interaction network

**Fig 1:**
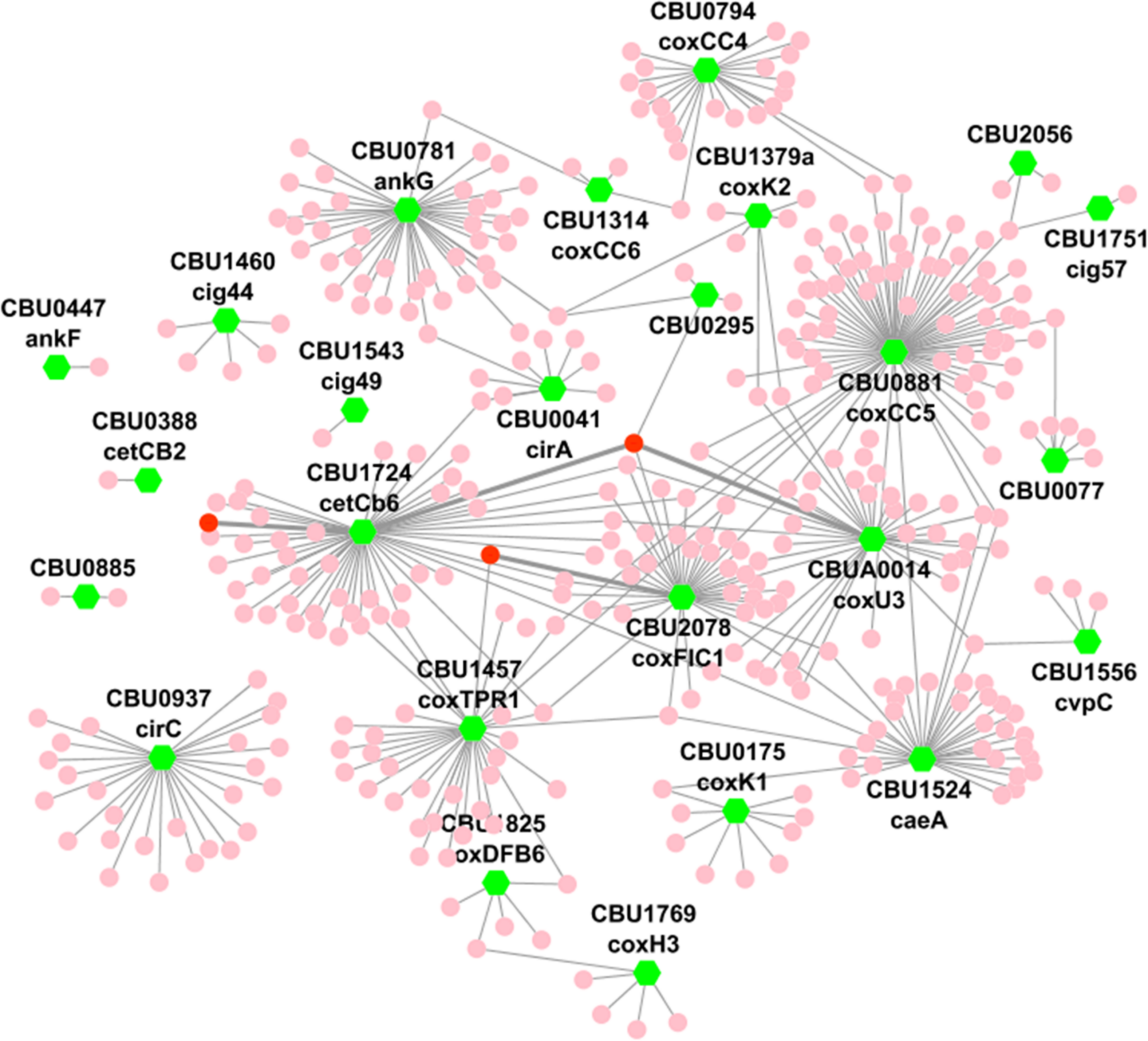
Yeast two-hybrid (Y2H) host-pathogen protein-protein interactions. Using Y2H screens against whole human and murine proteome libraries, we detected 273 unique interactions between 20 *Coxiella burnetii* and 247 human proteins and 157 unique interactions between 17 *C*. *burnetii* and 137 murine proteins. We used these data to construct a single host-pathogen protein interaction network, based on murine/human orthology, containing 415 unique interactions between 25 *C*. *burnetii* and 363 human proteins. Green nodes represent *C*. *burnetii* proteins, whereas pink and red nodes represent host proteins. Twelve *C*. *burnetii* proteins interacted with both hosts, three of which participated in conserved interactions (i.e., they interacted with both human proteins and their murine orthologs; shown as red nodes and connected with thick grey edges).

The set of 25 interacting proteins represents a large fraction of potentially important effectors with a role in establishing the *Coxiella* infection. The total set of all interactions represents an interaction profile of multiple pathogen-targeted processes used to establish and maintain the bacterial infection. To characterize this interaction profile, we merged the human-*C*. *burnetii* experimental and orthologous data sets to create an expanded set of human-*C*. *burnetii* pairwise PPIs consisting of 415 unique interactions between 25 *C*. *burnetii* and 363 human proteins. Fig 1 graphically shows the resulting host-pathogen protein interaction network, with all interactions provided in S2 Table.

### Protein domain analysis of targeted host proteins

We investigated the presence of conserved protein domains in host genes for nine *C*. *burnetii* effectors that interacted with 10 or more host proteins. Overall, 19 statistically over-represented conserved domains were identified among the effector-interacting host proteins. Table 2 shows that each effector has at least one statistically significant domain over-represented in the protein set compared to all host proteins, with over-represented host-domains typically present in 15% or less of the targeted host proteins for each effector. This indicated that no single domain dominated the effector-host protein binding. The major exception to this was the calcium-binding EGF-like domain (EGF_CA), which appeared in 26 host proteins targeted by CBUA0014 and in 12 targeted by CBU0781. This domain is present in a large number of membrane-bound proteins as well as extracellular proteins and is essential for numerous protein-protein interactions [44]. Furthermore, CBUA0014 also targeted two host domains—the PDZ_signaling domain among 10 host proteins, and the TGF-beta binding (TB) domain among nine host proteins. These domains are commonly identified as being part of multiple host protein-protein interaction events in organizing signaling complexes at cellular membranes that regulate cell proliferation, differentiation, and growth.

**Table 2:** Conserved domains among the *Coxiella burnetii*-interacting host genes.

Gene ID	Domain ID^1^	Domain Summary Description	N_d_^2^	N_h_^3^	N_db_^4^	*p*_raw_^5^	*p*_adj_^6^
CBU0781	zf-BED	Zinc finger DNA-binding domain in chromatin-boundary-element-binding proteins and transposases	4	40	5	3.0 10^−9^	1.3 10^−8^
	TB	TGF-beta binding (TB) domain; cysteine-rich repeat found in TGF-binding protein and fibrillin	3	40	38	1.1 10^−4^	1.3 10^−4^
	EGF_CA	Calcium-binding EGF-like domain; present in a large number of membrane-bound proteins, important in protein-protein interactions	12	40	514	9.6 10^−9^	3.1 10^−8^
CBU0794	zf-H2C2_2	Zinc-finger double domain	3	22	490	2.4 10^−2^	2.6 10^−2^
	zf-C2H2	Classic zinc-finger domain, associated with DNA- or protein- binding structural motifs, such as in eukaryotic transcription factors	3	22	234	3.4 10^−3^	3.9 10^−3^
CBU0881	BRCT	Breast cancer suppression protein (BRAC1) carboxy-terminal domain found predominantly in proteins involved in cell cycle checkpoint functions responsive to DNA damage	6	75	29	7.0 10^−9^	2.6 10^−8^
	zf-FCS	Zinc-finger domain that can function as a transcriptional trans-activator	8	75	26	1.5 10^−12^	9.5 10^−12^
	WEPRS_RNA	Domain involved in both protein-RNA interactions (by binding tRNA) and protein-protein interactions	3	75	4	2.3 10^−6^	3.2 10^−6^
	TSP_3	Thrombospondin type 3 repeat, containing short aspartate-rich repeats, which binds to calcium ions	3	75	14	4.3 10^−5^	5.5 10^−6^
	Cupredoxin	Domains that contain type I copper centers and are involved in inter-molecular electron transfer reactions	3	75	7	7.7 10^−6^	1.1 10^−5^
CBU0937	Calpain_inhib	Domain found in protein inhibitors of calpains, i.e., calcium-dependent, non-lysosomal cysteine proteases	3	26	3	6.3 10^−8^	1.8 10^−7^
CBU1457	Peptidase_M14NE-CP-C_like	C-terminal domain of M14 N/E carboxypeptidase; putative folding, regulation, or interaction domain	3	33	12	2.8 10^−6^	4.5 10^−6^
	Int_alpha	Integrin alpha (beta-propeller repeats); found in adhesion molecules that mediate cell-extracellular matrix and cell-cell interactions	5	33	74	4.7 10^−7^	1.1 10^−6^
	Collagen	Collagen triple helix repeat; found in structural proteins involved in formation of connective tissue structure	6	33	234	8.0 10^−7^	1.1 10^−6^
	RyR	Ryanodine receptor domain with unknown function	4	54	12	1.3 10^−7^	3.5 10^−7^
CBU2078	Int_alpha	Integrin alpha (beta-propeller repeats); found in adhesion molecules that mediate cell-extracellular matrix and cell-cell interactions	5	42	74	1.4 10^−6^	2.8 10^−7^
CBUA0014	vWFA	von Willebrand factor type A (vWA) domain, involved in basal membrane formation, cell migration, cell differentiation, adhesion, hemostasis, signaling, chromosomal stability, malignant transformation, and immune defenses	4	33	50	3.5 10^−6^	5.4 10^−6^
	PDZ_signaling	PDZ domain responsible for specific protein-protein interactions	10	33	197	2.3 10^−11^	1.0 10^−10^
	TB	TGF-beta binding (TB) domain; cysteine-rich repeat found in TGF-binding protein and fibrillin	9	33	38	6.1 10^−16^	7.9 10^−15^
	PDZ	PDZ domain that may play a role in scaffolding supramolecular complexes and in diverse signaling proteins	3	33	68	3.2 10^−4^	3.8 10^−3^
	FF	Involved in protein-protein interactions and in the regulation of actin cytoskeleton dynamics	3	33	10	1.7 10^−6^	3.2 10^−6^
	EGF_CA	Calcium-binding EGF-like domain; present in a large number of membrane-bound proteins, important in protein-protein interactions	26	33	514	2.6 10^−25^	6.7 10^−24^

^1^We performed domain identification, using NCBI CD Search (https://www.ncbi.nlm.nih.gov/Structure/bwrpsb/bwrpsb.cgi?) with default parameter settings for nine *Coxiella burnetii* (CB) genes that interacted with at least 10 host genes.

^2^N_d_: number of occurrences of the domain among the targeted proteins; only domains that occurred at least three times in the host genes were analyzed.

^3^N_h_: number of host genes that have the domain.

^4^N_db_: number of occurrences of the domain in the background set. All representative human proteins that have been manually reviewed were downloaded from UniProt (http://www.uniprot.org/). Among the 20,201 genes, we used the 19,036 genes containing domains (NCBI CD Search) as the background set for the statistical analyses.

^5^Original p-value from Fisher’s exact test.

^6^Adjusted p-value using the Benjamini–Hochberg correction procedure.

### Enrichment analysis of high-throughput Y2H host-pathogen interactions

The relatively large number of known effectors studied and host-protein interactions retrieved allowed us to analyze the combined interaction data as an “effector profile” of coordinated interactions to identify a potential repertoire of host functions targeted by *C*. *burnetii*. This approach was intended to generate broad hypotheses on common underlying effector mechanisms. Hence, we used all available high-throughput Y2H data to identify pathways, biological processes, and cellular locations associated with the targeted host proteins.

Table 3 shows the enriched KEGG pathways targeted by the effectors, using the orthology-derived high-throughput human-*C*. *burnetii* protein interaction network. This analysis indicates that the bacterial effectors have a preferential association with protein processing in the endoplasmic reticulum (ER), focal adhesion, and interference with protein degradation via the ubiquitin-proteasome pathway. The targeted host proteins affect glycolipid metabolism by interacting with host metabolism pathways involving small-branched hydrophobic amino acids. Finally, the effectors targeted host proteins involved in signaling via kinase regulation in the TGF-β and PI3K-AKT pathways.

**Table 3:** Enrichment of KEGG terms for human proteins interacting with *C*. *burnetii*.

**Pathway description**	***Cb*-targeted host proteins**	**KEGG proteins**	***p***_**raw**_	***p***_**adj**_
Protein processing in endoplasmic reticulum	11	85	2.8 10^−6^	3.3 10^−4^
TGF-β signaling pathway	7	43	4.6 10^−5^	3.5 10^−3^
Focal adhesion	7	60	4.0 10^−4^	0.02
Proteasome	6	43	4.0 10^−4^	0.02
Valine, leucine, and isoleucine degradation	5	29	4.6 10^−4^	0.02
Glycerolipid metabolism	6	16	2.6 10^−4^	0.02
PI3K-AKT signaling pathway	8	82	5.3 10^−4^	0.02

*Cb*, *Coxiella burnetii*; KEGG, Kyoto Encyclopedia of Genes and Genomes; *p*_raw_, original *p*-value; *p*_adj_, *p*-value adjusted according to the Benjamini-Hochberg multiple test correction [43].

Table 4 shows the enriched GO Biological Process terms associated with the host proteins identified by the Y2H screen of the 25 interacting bacterial proteins. These terms are largely compatible with the KEGG analysis in Table 3, and highlight specific processes associated with metabolism and ubiquitin processing. The GO analysis also highlights immune response modulation processes involving immune receptor signaling and antigen presentation processes. Additionally targeted host functionalities include posttranscriptional regulation of gene expression.

**Table 4:** Enrichment of GO Biological Process terms for human proteins interacting with *C*. *burnetii*.

Term description	*Cb*-targeted host proteins	GO proteins	*p*_raw_	*p*_adj_
*Metabolism*				
Nitrogen compound metabolic process	141	5759	4.2 10^−4^	0.10
Regulation of cellular amide metabolic process	16	311	4.5 10^−4^	0.10
*Immune response modulation*				
Fc-ε receptor signaling pathway	11	118	2.1 10^−5^	0.06
Stimulatory C-type lectin receptor signaling pathway	9	92	8.1 10^−5^	0.09
Antigen processing and presentation of exogenous antigen	11	156	2.6 10^−4^	0.10
*Ubiquitin processing*				
Positive regulation of ubiquitin-protease ligase activity	7	67	3.3 10^−4^	0.10
Regulation of protein ubiquitination	13	229	6.2 10^−4^	0.11
*Other*				
Posttranscriptional regulation of gene expression	21	392	3.3 10^−5^	0.06
Regulation of translation	15	283	5.0 10^−4^	0.11

*Cb*, *Coxiella burnetii*; GO, Gene Ontology; *p*_raw_, original *p*-value; *p*_adj_, adjusted *p*-value according to the Benjamini-Hochberg multiple test correction [43].

Table 5 shows the cellular localization of the host proteins. Overall, they were located in multiple cellular compartments in the cytoplasm, membrane-bound organelles, and nucleus. We could not identify any specific sub-nuclear location or particular intracellular vesicle for the host-targeted proteins, suggesting that they are present at multiple sites and potentially involved in multiple processes. The large number of host targets associated with extracellular exosomes points to processes associated with vesicles in general, as well as those with the potential to interact with the content of the exosomes as a means to influence the host immune response [45]. The locations associated with focal adhesions, adherence junctions, and microtubule cytoskeletons point to a preference for influencing cell signaling and vacuolar re-arrangements associated with the infection. The association of targeted proteins with the ribonucleoprotein complex is consistent with a potential role for effectors in interfering with host protein processing in the ER. Additionally, a number of targeted proteins were located in the mitochondrial matrix.

**Table 5:** Enrichment of GO Cellular Component terms for human proteins interacting with *C*. *burnetii*.

**Term description**	***Cb*-targeted host proteins**	**GO proteins**	***p***_**raw**_	***p***_**adj**_
*Overall location*				
Cytoplasm	221	9158	4.3∙10^−10^	3.0∙10^−8^
Membrane-bound organelle	238	10322	2.1∙10^−9^	1.1∙10^−7^
Nucleus	147	5873	6.1∙10^−6^	2.0∙10^−4^
*Specific location*				
Extracellular exosome	88	2427	2.0∙10^−10^	1.8∙10^−8^
Vesicle	106	3245	5.8∙10^−10^	3.6∙10^−8^
Ribonucleoprotein complex	24	603	4.5∙10^−4^	8.2∙10^−3^
Focal adhesion	15	334	1.6∙10^−3^	0.02
Mitochondrial matrix	15	346	2.3∙10^−3^	0.03
Adherence junction	16	400	3.8∙10^−4^	0.05
ESCRT complex	11	218	4.2∙10^−4^	0.06
Microtubule organizing center	19	534	5.9∙10^−3^	0.06
ER-Golgi compartment	5	82	0.02	0.13

*Cb*, *Coxiella burnetii*; ER, endoplasmic reticulum; ESCRT, endosomal sorting complexes required for transport; GO, Gene Ontology; *p*_raw_, original *p*-value; *p*_adj_, adjusted *p*-value according to the Benjamini-Hochberg multiple test correction [43].

### Emerging interaction patterns from patterns of host interactions

*Coxiella* has the capability to successfully infect different eukaryotic hosts and cell types by using sets of translocated T4SS effector proteins. This implies that the interactions may be non-specific yet concerted to establish the biological host phenotype amenable to pathogen survival. The mechanisms by which *Coxiella* manipulates host cell processes are largely unknown, but effector-targeted host proteins can provide a mechanistic understanding of pathogenesis.

Our pathway analysis identified a number of metabolic host target proteins involved in small hydrophobic amino acid degradation and glycerolipid metabolism (Table 3). Given that metabolism has not been considered as a host target process of T4SS effectors before [46], this suggests novel mechanisms involving energy metabolism (triglycerides are primarily used for energy storage) and essential amino acids (valine, leucine, and isoleucine), which may be related to preventing host cell autophagy by blocking a host-defensive starvation response [47].

**Fig 2:**
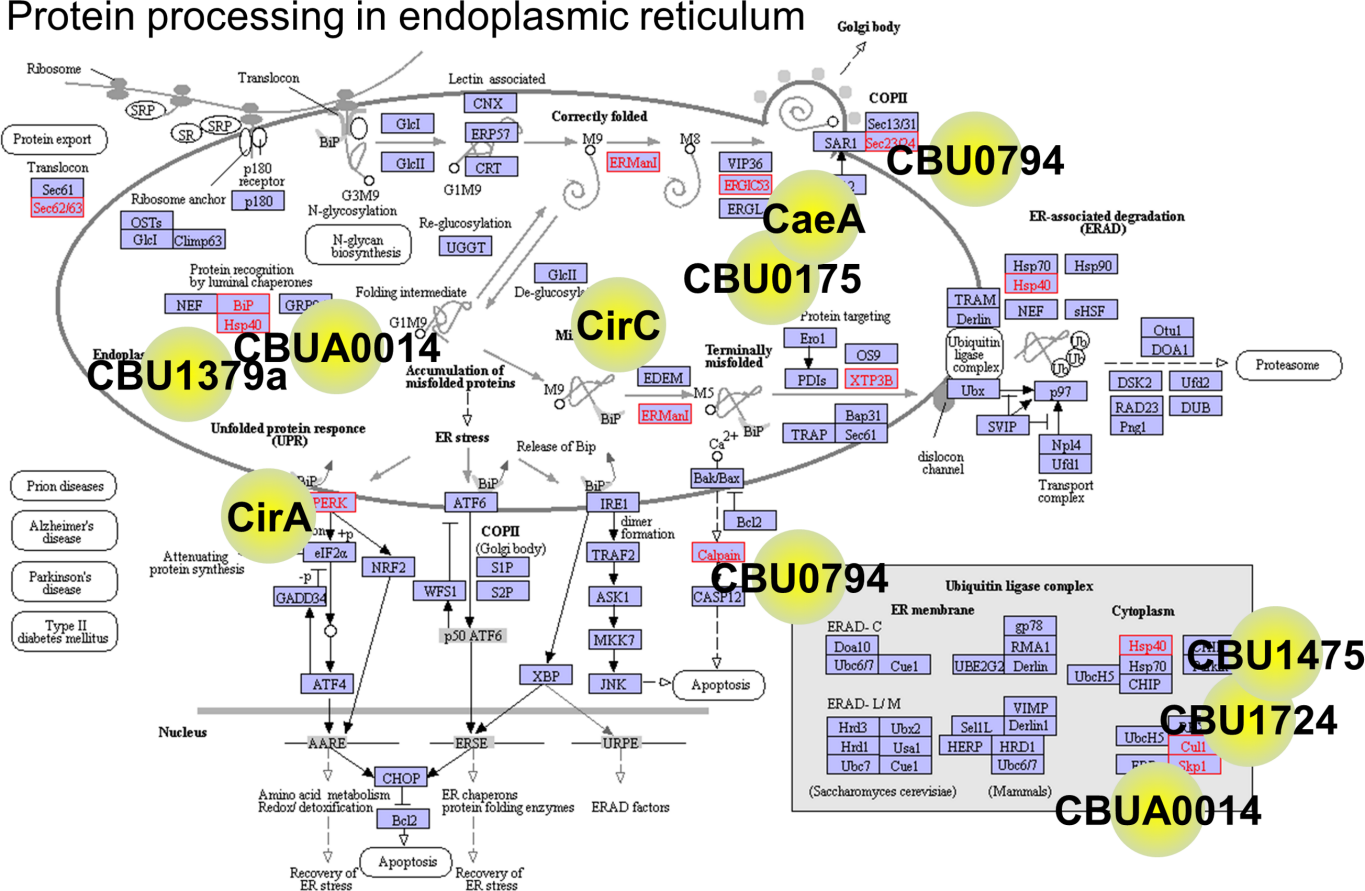
*Coxiella* interactions in the endoplasmic reticulum protein-processing pathway. The interacting host proteins are highlighted in red text and the pathogen proteins are superimposed as named yellow circles and located close to their interacting host partners. Overlaying the interacting *C*. *burnetii* proteins onto their human partners in Kyoto Encyclopedia of Genes and Genomes (KEGG) pathways illustrates intervention points of nine screened *Coxiella* effectors that could affect human-host protein processing in the endoplasmic reticulum (ER). Pathogen proteins can multiply their effect by interacting with multiple host proteins or focus their effect by using different pathogen proteins to target the same host protein. The potential effect of these interactions could influence multiple processes, such as protein export, COPII-mediated vesicle formation, initiation of apoptosis, and ER-assisted protein degradation. In this pathway representation, CBUA0014 interacts with Hsp40 and Skp1; CBU1379a with BiP; CirA with PERK; CirC with ERManI; CBU0794 with Calpain and Sec23/24; CBU1724 with Cul1; and CBU1457 with Cul1. The underlying network graph is reprinted from KEGG [40] under a CC BY license, with permission from the Kanehisa Laboratories, original copyright 2016.

The ability of *C*. *burnetii* to orchestrate physiological processes of host-cell organelles and interfere with host protein processing was evident from the large number of protein binding events that preferentially could take place in the ER and Golgi (Tables 3–5). Fig 2 shows the intervention points of nine screened *Coxiella* effectors affecting human-host protein processing in the ER. Previous studies have noted the importance and occurrence of pathogen interactions with the ER as a critical component of lipid metabolism, protein synthesis, protein trafficking, and cellular stress responses [48, 49]. Fig 2 illustrates how both a single effector (CBU0794) interacted with multiple host proteins as well as multiple pathogen proteins targeting individual host proteins (Hsp40 and ERManI). The coordinated interactions affected processes, such as protein export, COPII-mediated vesicle formation, initiation of apoptosis, and ER-assisted degradation, via the ubiquitin-proteasome system. The recently noted interactions between the CCV and the ER, which involve the host protein oxysterol-binding protein homologue ORP1L and RAS oncogene RAB7A [49], were partly captured in our data with CBU0794 interacting with the oxysterol binding protein OSBPL1A.

The summaries of host protein interactions in Tables 3–5 implicated multiple coupled host pathways involving focal adhesion, immune signaling and response (primarily via ubiquitin-proteasome degradation), and changes in cell morphology. The main host targets in the focal adhesion pathway involve MAPK signaling pathways regulating cell survival and the ability to modulate the actin cytoskeleton and cell morphology [50–54]. Kinase interactions involved CBU1724 binding with MAPK10 to modulate cellular stress responses. The remaining effectors targeting the focal adhesion pathway were linked to proteins that regulate actin cytoskeletal remodeling of cell morphology and cell-cell or cell-matrix interactions. The ability to regulate or modulate actin cytoskeleton remodeling in altering cell morphology is also linked to interference in the transitions between phases of the cell cycle, with the polarization state of alveolar macrophages influencing the susceptibility of the host cells to infection [55]. *C*. *burnetii* interactions at centrosome microtubules could serve to regulate cell cycle progression by interfering with or altering spindle assembly and, thereby, changing cell polarity. The host targets for these interactions include both regulatory elements, such as ROCK1 and RBL2, as well as the actin-binding protein ANLN.

Pathogen-host interactions that interfered with different components of the immune system involved both a signaling component directly targeting the Fc receptor (FCER1G). Although antibody-mediated immunity to *C*. *burnetii* is thought to be Fc receptor independent [56], the ability to directly bind to the receptor may have cascading effects interfering with phagocytosis and cytokine generation. The ubiquitin-proteasome system was affected by both ubiquitin-ligase complex interactions as well as by numerous proteasome sub-unit interactions involving multiple *C*. *burnetii* proteins. Modulation of the ubiquitin-proteasome pathway is a feature of the strategy utilized by the closely related pathogen *Legionella pneumophila* [57, 58], which co-opts the host protein degradation system to temporally regulate bacterial effectors in the host cell.

The preferential cellular locations of targeted host proteins (Table 5) also point to important bacterial mechanisms related to endosomal trafficking, vacuole creation, and exosome function [45,59,60]. They indicated that a large number of pathogen targets associated with extracellular host exosomes form a broad group of *C*. *burnetii*-targeted proteins; almost 30% of all protein interactions could be linked to exosomes. Although the targeted host proteins are involved in numerous physiological functions, their main roles are in proteolysis via proteasome interactions, cytoskeletal arrangement via actin regulation, chaperone activity through heat-shock proteins, and oxidative stress responses via thioredoxin interactions. The main function of extracellular exosomes is to enable intercellular host communication, primarily as carriers of immune signals during pathogen infections. For example, Salmonella-infected host cells secrete exosomes enriched in bacterial lipopolysaccharides [61]. The potential ability of *C*. *burnetii* to interfere with this process is a novel aspect of the immune evasion by this pathogen.

Proteins linked to endosomal sorting complexes required for transport (ESCRT) also function in vesicle budding, a process used by the uninfected host cell to control membrane abscission during cytokinesis. Intracellular vacuolar pathogens, such as *Salmonella*, *Legionella*, and *Coxiella*, must be able to influence these key host processes to establish their specialized intracellular environments. The interaction set targeting the ESCRT involved 7 pathogen proteins and 11 host proteins associated with 17 total host-pathogen interactions. Of special note is the ER to Golgi protein trafficking host protein TRAPPC8 [62], which potentially interacts with four different *C*. *burnetii* effectors; ubiquitin peptidase USP8, which regulates endosome morphology and protein transport [63], and NPC2, which is involved in cholesterol trafficking [64].

The identification of the mitochondrial matrix as a host location targeted by *C*. *burnetii* effectors implicated multiple processes that may lead to a more complex mitochondrial phenotype than just inhibition of apoptosis [65–67]. Of the 16 host-pathogen protein interactions among 14 host proteins and 11 *C*. *burnetii* proteins, 31% were linked to protein synthesis within mitochondria, 19% to fatty acid beta-oxidation, with the rest involving different enzymatic catabolic functions.

**Fig 3:**
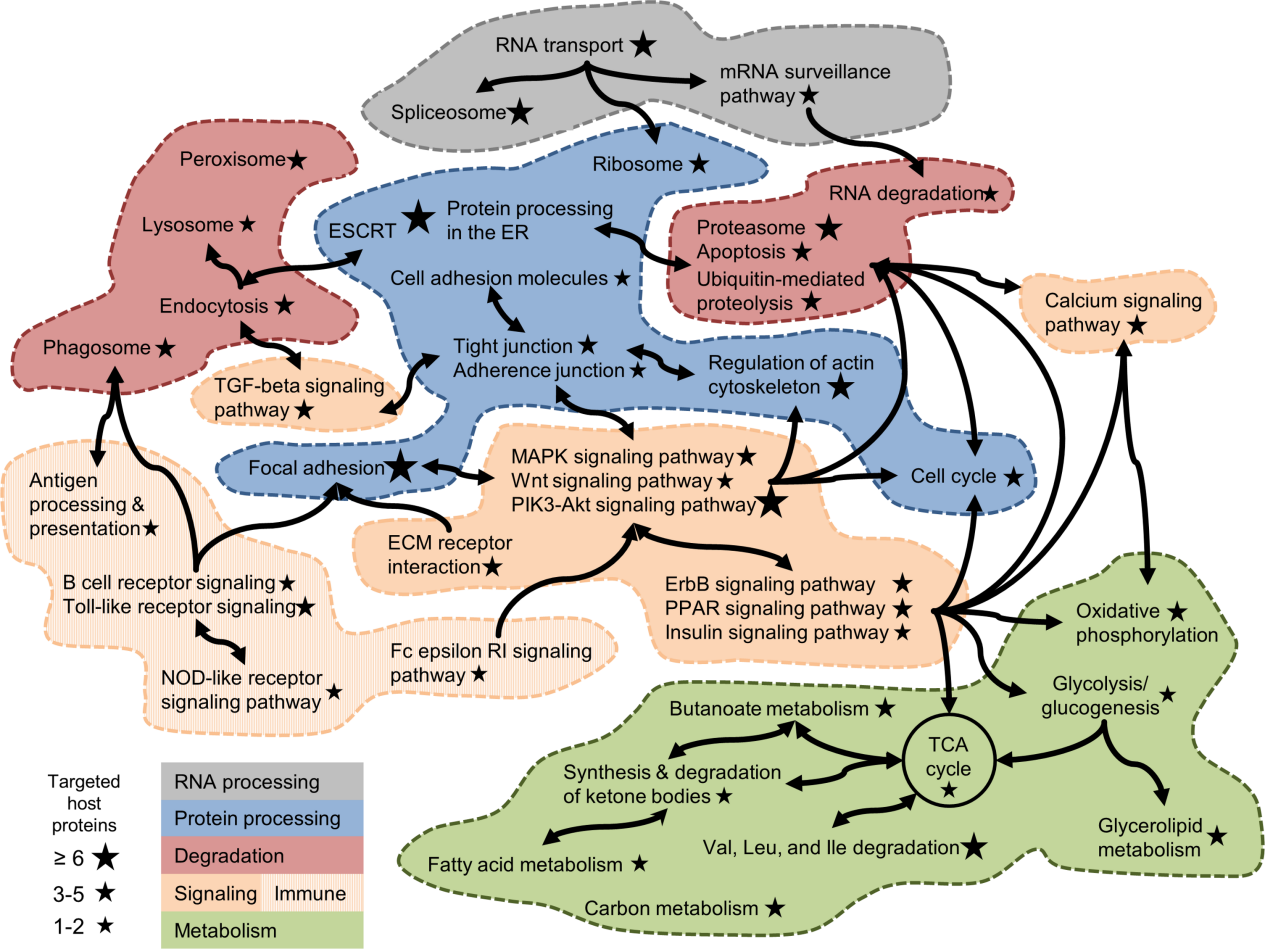
Host pathways targeted by *Coxiella*. *C*. *burnetii*-interacting host proteins are present in interconnected Kyoto Encyclopedia of Genes and Genomes (KEGG) pathways with the potential to affect multiple cellular processes of the host. The pathways are grouped into five major categories: RNA processing, protein processing, degradation pathways, signaling (including signaling events related to the immune response), and metabolism. The size of a star indicates the number of targeted host proteins in each pathway. ECM, extracellular matrix; ER, endoplasmic reticulum; ErbB, erythroblastic leukemia viral oncogene; ESCRT, endosomal sorting complexes required for transport; MAPK, mitogen-activated protein kinase; NOD, nucleotide-binding oligomerization domain; PIK3, phosphatidylinositol-3-kinases; TCA, tricarboxylic acid; TGF, transforming growth factor.

The presence of multiple host targets in different pathways points to a possible higher biological or casual organization of bacterial effector interactions. Fig 3 shows the interconnected nature of *Coxiella*-targeted host pathways according to the number of pathogen-interacting host proteins present in human KEGG pathways. We have broadly arranged and grouped these pathways to highlight RNA processing, protein handling, macromolecular degradation, cellular signaling (including immune-related signaling), and metabolism. Pathway intersections (crosstalk) are indicated by arrows and highlight the potential of effector proteins to affect multiple cellular processes of the host. Although pathway enrichment analysis highlights the presence of multiple targets, each individual interaction could also be critical to the function of the pathway or have physiologically important downstream effects. The biological assumption underlying pathway enrichment analysis is that by affecting multiple targets in a pathway, there is a higher chance that the function of a pathway may be altered. Hence, a bacterium that has become successful at infecting hosts is assumed to have developed a higher propensity to affect multiple proteins in that pathway. Because this may not always be the case, the interpretation of the interaction data should also consider the overall broader set of potentially affected pathways as well as individual sets of effector host-pathogen interactions. The pathway mapping in Fig 3 links major immune and stress signaling pathways to *1*) cellular remodeling pathways, *2*) different lytic vesicles, proteolysis, and programmed cell death, and *3*) metabolic pathways involved in amino acid degradation and energy production.

The aggregate information derived from all host-pathogen interactions represents a spatial and temporal average of these interactions that do not capture infection progression or maintenance *per se*. What emerges from the interaction profile is a broad pattern of host process interference, which is commensurate with known *Coxiella/Legionella* infections and strongly emphasizes the ability to affect host organelle creation, endosomal trafficking, and organelle interactions with the parasitophorous vacuole [68–72].

### Using Y2H interactions to characterize individual *C*. *burnetii* effector function

We used the high-throughput Y2H interactions to identify processes and pathways with a high probability of being affected during the infection. The underlying assumption of this analysis—that multiple protein interactions exert a coordinated effect to re-direct host processes—is based partly on the observation that the bacterium is able to non-specifically infect multiple hosts and cell types and partly on the finding that a large number of effectors are secreted into the host cell. Thus, although some individual interactions may be incorrect, the broader integration patterns of coordinated action should still be evident in these analyses. Conversely, we also used these interactions in conjunction with a smaller pairwise test set to better characterize a set of four effectors for which evidence on their functional significance is lacking.

### Complementary pairwise Y2H testing

We carried out high-throughput pathogen-host protein interaction screening using *C*. *burnetii* ORFs and human and murine random cDNA libraries. In order to assess the feasibility of using the well-characterized human ORFeome resources to validate the high-throughput Y2H interaction, we selected as subset of *C*. *burnetii*-human PPIs and tested them using pairwise Y2H screening. Herein, the Y2H prey clones were generated by cloning the human ORFeome clones to pGADT7g [37], a modified Gateway technology-compatible Y2H prey vector. This prey vector differs in the linker amino acid sequences encoded between the activation domain and the ORFs. Furthermore, the human ORFeome clones do not contain an endogenous stop codon; hence, they encode additional peptide sequences at the C-terminal ends of the ORFs, which may affect their interaction patterns. Previous studies have shown that such differences in either bait or prey vectors produce significantly different interaction data [33, 73]. Our expectation was to reproduce approximately 50% of interactions in the pairwise Y2H screening, as minor variations in two-hybrid vectors detect markedly different subsets of interactions in the same interactome space [74].

In this pairwise low-throughput Y2H screen, we individually tested selected interactions involving four relatively uncharacterized *C*. *burnetii* effector proteins plus interactions associated with the plasmid CBUA0014 effector examined in the high-throughput screens. Thus, the pairwise testing encompassed 91 host-protein interactions from five *C*. *burnetii* proteins—CBU0794, CBU0881, CBU1724, CBU2078, and CBUA0014—which involved 22, 75, 54, 42, and 33 total interactions in the high-throughput screen, respectively. Briefly, of the 226 individual interactions found in the high-throughput screen for these effectors, we constructed Y2H clones and tested 91 or 40% in a pairwise Y2H assay. Of these, 43 (47%) tested positive. There was no correlation between the fraction of interactions that tested positive and the number of interactions in the high-throughput screen, which further suggests that interactions not seen in the pairwise Y2H screening could be due to the intrinsic variation in the vectors and cDNA clone used. The average interaction reproducibility per effector of 47% with a sample standard deviation of 19% was better than, or on par, with other experimental studies [34, 75–77]. All individual results are included in S2 Table.

### Individual *C*. *burnetii* effector host-pathogen interactions

Table 6 lists known effectors, with known mechanistic associations, which we tested in our high-throughput Y2H screens. Given their importance as pathogen proteins critical for *C*. *burnetii* infections, we used the Y2H data to examine both location and functional annotations associated with the targeted host proteins. Table 6 provides their common names, keywords from published literature data, and a summary of the interaction analyses—the number of host interactions, top four candidate cellular locations of interacting host proteins, direct functional evidence from individual host protein interactions, and enriched pathways/locations (Tables 3–5), including host-pathogen interactions where the effector took part. Note that the sum of the fractions of protein locations can exceed one, due to the possible presence of proteins in multiple locations, e.g., if all targeted proteins were present both in the nucleus and in mitochondria, both location fractions would be 100%.

**Table 6:** Functional characterization based on host-protein interaction data.

Locus	Name	Keywords	Y2H protein interaction data						
			N_host_	Top locations (%)				Individual interactions	Pathways
CBU0781	ankG	anti-apoptotic; enters nucleus	40{	Nucleus	Exosome	Vesicle	Mito	apoptotic control; cell cycle	Fc receptor pathway; mitochondrial matrix
				(50)	(33)	(32)	(10)		
CBU0937	cirC	central for intracellular replication	26{	Nucleus	Exosome	Mito	Memb	stress response, solute transport	Fc receptor pathway; mitochondrial matrix
				(38)	(23)	(15)	(15)		
CBU1314	-	nuclear effector; modulation of host transcriptome	4{	Nucleus	Exosome	-	-	proteasome complex	focal adhesion; NF-κB immune-response
				(75)	(75)				
CBU1524	caeA	anti-apoptotic; enters nucleus	35{	Nucleus	Exosome	ER/Golg	Memb	spliceosome; ER-assisted protein degradation	endosomal sorting; NF-κB immune-response; cell cycle phase transition
				(65)	(46)	(17)	(6)		
CBUA0014	cepC	E3 ubiquitin ligase binding	32{	Nucleus	Exosome	Memb	ER/Golg	pleiotropic, ubiquitination, vacuolar sorting	endosomal sorting; Fc receptor pathway; focal adhesion; mitochondrial matrix; NF-κB immune-response
				(41)	(25)	(18)	(12)		

ER/Golg, endoplasmic reticulum/Golgi apparatus; Memb, plasma membrane; Mito, mitochondria; N_host_, number of host-pathogen protein interactions; Y2H, yeast two-hybrid.

The T4SS effector CBU0781 (ankG) is linked to modulation/prevention of intrinsic host cell apoptosis, located in mitochondria, and subsequently translocated to the nucleus [17, 77,78,79]. Of the 40 identified host targets, the four proteins localized to mitochondria (BBOX1, DLAT, ECH1, and OCIAD21) are involved in metabolism. Most (50%) of the targeted host proteins are located in the nucleus, with the remainder primarily located at sites associated with the plasma membrane or different membrane-bound cellular organelles. Of note is the observed Y2H interaction between ankG and KPNA3, the importin subunit α3 of the nuclear pore complex, which is required for the localization of ankG to the nucleus [17]. The ankG-host protein interactions in the nucleus are associated with apoptotic processes involving ABCA1 [80], PARP4 [81], and PGRMC2 [82], as well as interference in cell cycle/mitotic progression via CDK14 [83], STAG3 [84], RFX5 [85], and BAZ2B [86].

The CBU0937 (cirC) effector was determined to be an immunoreactive Q fever-specific protein [87] and important for intracellular replication. Examination of the host protein targets did not unequivocally identify a virulence mechanism associated with this effector. The primary locations of the host proteins indicated multiple potential sites and mechanisms. Protein targets in the plasma membrane may be related to solute transport and calcium and cationic exchange, whereas those located in mitochondria could be linked to mitochondrial protein synthesis. Targets in the nucleus could be related to stress responses (MAP2K4, PPM1G, and RGS2) and RNA processing (PA2G4 and PAPOLA).

The nuclear effector CBU1314 modulates the host transcription response [88], but with scant Y2H interaction data we could only identify nuclear host protein interactions related to focal adhesion (the Rho GTPase activating ARHGAP5 gene) and proteasome subunit interactions involved in the NF-κB immune-response pathway.

CBU1524 (caeA) is linked to intranuclear interactions that prevent or delay apoptosis of the host cell [66,67,89]. Most (65%) targeted host proteins were located in the nucleus, consistent with the finding that caeA is preferentially located in the nucleus [89]. However, substantial fractions were also observed in the plasma membrane (25%) and in the ER/Golgi apparatus (17%). The roles of the targeted nuclear proteins related to different aspects of gene translation, such as splicing (e.g., spliceosome-associated proteins DHX15, SAP18, and SNRPA1), RNA transport, and RNA transcription, as well as ubiquitin-proteasome regulation. Those of the host proteins localized to ER sites were associated with specific ER-associated protein degradation (ERLIN2) and apoptosis (PKD2 [90] and RTN4 [91,92]).

We also investigated the potential interactions of CBU0077, which accumulates at vacuolar membranes and abnormal ER extensions, suggesting that it interferes with vesicular traffic [89]. Recent research tag CBU0077 as a complex-forming effector at the mitochondrial outer membrane during Coxiella infection [93]. Six host proteins interacted with CBU0077, three of which directly related to the reported phenotype (i.e., the junction adhesion molecule AMICA1 [94], the transmembrane-trafficking protein TMED10, and ACADM as part of the mitochondrial fatty acid beta-oxidation pathway. None of these host proteins were located in lysosomes, which are the preferred sites in HeLa cells [30], indicating that the roles of these proteins may vary in different cell types [69].

The presence of effectors on the conserved *Coxiella* plasmid indicates the importance of their roles. The plasmid effector CBUA0014 (cpeC), an F-box homology protein, localizes to ubiquitin-rich compartments and is hypothesized to play an important role in establishing host infections [95]. Table 6 shows a range of cellular sites where potential host target proteins are preferentially located (nucleus, ER/Golgi apparatus, and plasma membrane), as well as several specific pathways that may be impacted by this effector, including components of the ubiquitin-proteasome system and vacuolar sorting pathway (retromer complexes). Of note are the protein interactions we verified in the complementary pairwise Y2H testing experiment. Thus, cpeC could bind to the ubiquitin-ligase complex component SKP1, which is part of the SKP1-CUL1-F-box protein complex, as corroborated by the observation that ubiquitin co-localizes with this effector [95]. The potential pleiotropic role of cpeC is indicated by the verified Y2H interactions with VPS26A, which is involved in protein sorting of vacuolar pathogens and TMX2 [96] as part of the ESCRT endosomal sorting complex.

### Inferring mechanisms of action for relatively uncharacterized effectors

We further selected four additional effectors (CBU0794, CBU0881, CBU1724, and CBU2078) with little or no information on their role during infections for analysis and complementary pairwise Y2H testing, in an attempt to characterize their potential functions. Table 7 provides an overview of these protein interaction analyses, including alternate gene symbols, the success of the complementary testing effort for these proteins, the number of host interactions, the top four cellular locations where interacting host proteins may be located, direct functional evidence from individual host protein interactions, and a list of enriched pathways/locations from Tables 3–5 involving the effector.

**Table 7:** Unknown secreted effector characterization based on host-protein interaction data.

**Locus**	**Name**	Keywords	**Y2H protein interaction data**						
			N_host_	Top locations (%)				Individual interactions	Pathways
CBU0781	ankG	anti-apoptotic; enters nucleus	40{	Nucleus	Exosome	Vesicle	Mito	apoptotic control; cell cycle	Fc receptor pathway; mitochondrial matrix
				(50)	(33)	(32)	(10)		
CBU0937	cirC	central for intracellular replication	26{	Nucleus	Exosome	Mito	Memb	stress response, solute transport	Fc receptor pathway; mitochondrial matrix
				(38)	(23)	(15)	(15)		
CBU1314	-	nuclear effector; modulation of host transcriptome	4{	Nucleus	Exosome	-	-	proteasome complex	focal adhesion; NF-κB immune-response
				(75)	(75)				
CBU1524	caeA	anti-apoptotic; enters nucleus	35{	Nucleus	Exosome	ER/Golg	Memb	spliceosome; ER-assisted protein degradation	endosomal sorting; NF-κB immune-response; cell cycle phase transition
				(65)	(46)	(17)	(6)		
CBUA0014	cepC	E3 ubiquitin ligase binding	32{	Nucleus	Exosome	Memb	ER/Golg	pleiotropic, ubiquitination, vacuolar sorting	endosomal sorting; Fc receptor pathway; focal adhesion; mitochondrial matrix; NF-κB immune-response
				(41)	(25)	(18)	(12)		

Centro, centrosome; ER/Golg, endoplasmic reticulum/Golgi apparatus; ESCRT, endosomal sorting complexes required for transport; Memb, plasma membrane; Mito, mitochondria; N_host_, number of host-pathogen protein interactions; Y2H, yeast two-hybrid.

The hypothetical protein CBU0794 exhibited interactions with host proteins in the focal adhesion pathway (CAPN2) and with the proteasome (PSMC1), but the relatively low number of interactions and the low number of overall successfully pairwise tested Y2H interactions did not allow us to define a more precise role for this effector.

The potential effector CBU0881 exhibited the second largest number of host-pathogen interactions in our data set involving host pathways/processes, such as endosomal sorting, focal adhesion, the NF-κB immune-response, and protein processing in mitochondria. Complementary Y2H testing confirmed two interactions in mitochondria involving the peptidase PITRM1, which is responsible for degrading transit peptide after their cleavage, and the tRNA-synthetase IARS2, which is involved in mitochondrial protein synthesis. The interaction data also point to extensive interactions of CBU0881 with IST1, ATP6V1E2, CAPN7, and USP8 in the endosomal ESCRT pathway, with only the last interaction retested but not confirmed.

The complementary Y2H testing data confirmed several interactions associated with the centrosome, pointing to a heretofore unrecognized *Coxiella*-targeted organelle. The high-throughput screens identified a total of 14 host targets located in the centrosome, eight of which interacted with CBU0881. Of five tested interactions, we confirmed three in the pairwise Y2H screen: DCTN6, EFHC, and NEK2. The corresponding functional relationships associated with their centrosome location relate to protein organelle/vesicle transport and spindle morphogenesis (DCTN6 [97]), microtubule-regulation of cell division (EFHC1 [98]), and centrosome separation control and bipolar spindle formation (NEK2 [99,100]). Further indications of the potential pleiotropic role of this effector came from the confirmed interactions associated with nucleus-located host proteins involved in transcription via FAM60A in translational repression and the peptidylprolyl isomerase PPIE in the spliceosome.

CBU1724 interacted with 17 human and 37 mouse proteins with two overlapping interactions (Table 1). The pathway analyses indicated multiple roles in multiple pathways with selected interactions tested and all confirmed in these pathways (MAPK10, ANLN, AK3, and NPC2). Further evidence of a pleiotropic role for this protein was revealed by the interactions with proteins involved in ubiquitin handling and processing, such as CUL1, MYCBP2, OTUB1, and UBE2V2, with the interaction between CBU1724 and the ubiquitin thioesterase OTUB1 confirmed in the complementary testing.

In addition to the high-throughput screening identification of transthyretin (TTR) as a potential *C*. *burnetii* target involved in cholesterol processing [101], complementary testing confirmed two additional host proteins involved in cholesterol handling: the apolipoprotein APOA1BP [102] and the intracellular cholesterol transporter NPC2 [103]. Although *C*. *burnetii* infections require cholesterol from the host [64,104], pathogen mechanisms that influence cholesterol uptake and processing in the host are not well characterized. The use of T4SS effectors as potential mediators of this process would thus be critical for maintaining and propagating the infection.

For the final effector, the high-throughput screening pathway analysis and accompanying Y2H pairwise testing data revealed that CBU2078 interacts with host proteins involved in focal adhesion and endosomal sorting. Analysis of the complementary testing data confirmed interactions for four of seven proteins involved in the ubiquitin-proteasome system: RBBP6, RNF38, USP47, and WDR48. These proteins included ubiquitin ligase/proteases (RBBP6, RNF38, and USP47) as well as a protein that stimulates the activity of ubiquitin-specific proteases (WDR48) [105,106].

## Discussion

### Y2H characterization of bacterial effector proteins

Large-scale high-throughput Y2H protein interaction screens can yield large amounts of novel and unbiased data. Here, we used this technology to focus on a select set of secreted bacterial effectors in an effort to characterize *C*. *burnetii* host-pathogen interactions. We conducted the largest human and murine genome-scale Y2H screening to date, using 33 *C*. *burnetii* T4SS effectors to identify and characterize host-pathogen PPIs among this set. The aim of this study was thus to derive general hypotheses on the mechanisms of effector actions mediated by protein interactions. These screens provided partial host interaction data for 25 bacterial effectors distributed among 415 unique pairwise interactions.

Our analyses of these data and the hypotheses derived from studying individual and multiple interactions rely on the underlying protein interactions having biological significance. The nature of protein interactions is complex; high-affinity interactions are not necessarily more biologically relevant than low-affinity interactions because transient interactions with signaling proteins may have important downstream effects not directly captured by PPI data. Many of these low-affinity and transient interactions are strongly dependent on the physiological state of the cell and may vary with the intracellular environment. Similarly, host and pathogen proteins must be simultaneously present in the same cellular compartment and of sufficient quantity for the predicted interaction to be biologically relevant. Thus, although all T4SS effectors are initially released into the cytoplasm of the host cell, their primary targets may be host-cell compartment/organelle specific, and their effects may depend on the ability to efficiently translocate to the correct site of action at the appropriate time.

*C*. *burnetii* is capable of colonizing multiple eukaryotic hosts, including different mammalian species and many different cell types. Consequently, the basic infection program executed by the pathogen must exhibit both the generalized and specific features of host-pathway interactions. A corollary of this assumption is that the likelihood of each host-pathogen protein interaction being highly specific, involving strong binary interactions, or being essential for establishing the intracellular infection is small. Thus, we used the high-throughput screening data to identify the biological processes or pathways and cellular locations to which the targeted host proteins belong, in order to characterize aspects of the general impact on the host cell of the tested effectors.

### Pathways and processes targeted by *C*. *burnetii*

The pathway enrichment analysis of the interactions detected in the Y2H high-throughput screens identified host cellular processes known to occur in *Coxiella* infections and broadly compatible with both *Legionella* and *Salmonella* infection phenotypes. Not surprisingly, many of these related to cellular processes in which direct protein interactions are expected to play a critical role, such as in adapting cell morphology through cytoskeletal re-arrangements, protein processing and trafficking, and vacuolar generation. Similarly, direct protein interactions could strongly affect the frequently observed interactions with components of the ubiquitin-proteasome pathways. The enrichment analyses also indicated targeted host processes involved in amino acid metabolism and cholesterol processing, indicating a potential nutritional component associated with the infection phenotype and T4SS effectors. The interaction data also revealed the exosome to be a preferred location for many of the targeted host proteins. If *C*. *burnetii* effectors interfere with the intracellular trafficking of immature exosomes or interact with proteins in exosomes, they would be expected to dampen the immune signaling that occurs between host cells and prevent the host from mounting a more robust immune response.

We also examined the host proteins targeted by specific effectors previously found to alter *Coxiella* infection phenotypes in transposon mutant studies. We used the interaction data to characterize potential mechanisms of action and identify specific host targets for effectors that could be associated with the observed mutant phenotypes for ankG, cirC, and caeA. In the case of ankG and caeA, we used their nuclear locations in the host cell to focus the analysis on apoptotic control and interference with host gene transcription. In contrast, for other proteins such as CBU1314, the interaction data were limited, suggesting that protein interactions are not involved in their mode of operation.

Finally, we considered the interaction data to derive hypotheses on the potential mechanisms of action of four individual effectors for which phenotypic information is sparse. In three cases, the joint interaction data supported mechanisms that were broadly similar to those of the other effectors, such as involvement in ESCRT processes, ubiquitin handling, and transcriptional control; at the same time, they also pointed to specific processes not previously associated with T4SS effectors, such as cholesterol transport (CBU1724) and centrosome interactions (CBU0881). As characterizing novel effectors is often referred to as “the hard part” [2], careful *in vitro* and *in vivo* phenotypic studies of deletion mutants will be needed to acquire more precise information on their specific roles.

The present study delineates the largest collection thus far of *C*. *burnetii* effector protein interactions derived from whole-genome human and murine host protein libraries. This data set represents a rich source of information on secreted pathogen effector proteins and their interactions with host proteins. As such, these interactions contain critical information on how the pathogen establishes and maintains a successful intracellular infection.

## Supporting information

S1 Table Excluded host proteins. The table provides host proteins known to be indiscriminate binders and eliminated from our yeast two-hybrid high-throughput screen.
(XLSX)

S2 Table Host-pathogen interaction data. The table provides all pairwise interactions detected in the yeast two-hybrid (Y2H) high-throughput screen from the human libraries and the corresponding murine orthologous interactions as well as the pairwise interactions from the complementary pairwise Y2H testing.
(DOCX)

## Abbreviations

3-AT: 3-amino-1,2,4-triazole
ABCA1: ATP binding cassette subfamily A member 1
AK3: adenylate kinase 3
AMICA1: adhesion molecule, interacts with CXADR antigen 1
ANLN: anillin, actin binding protein
APOA1BP: apolipoprotein A-I-binding protein
ARHGAP5: Rho GTPase–activating protein 5
ATP6V1E2: ATPase H+ transporting V1 subunit E2
BAZ2B: bromodomain adjacent to zinc finger domain 2B
BBOX1: gamma-butyrobetaine hydroxylase 1
CAPN2: calpain 2
CAPN7: calpain 7
CDK14: cyclin dependent kinase 14
CUL1: cullin-1
DHX15: DEAH-box helicase 15
DLAT: dihydrolipoamide S-acetyltransferase
ECH1: enoyl-CoA hydratase 1
EFHC1: EF-hand domain-containing protein 1
ERLIN2: ER lipid raft associated 2
ESCRT: endosomal sorting complexes required for transport
FAM60A: family with sequence similarity 60 member A
FCER1G: Fc fragment of IgE receptor Ig
GO: Gene Ontology
IARS2: isoleucyl-tRNA synthetase 2, mitochondrial
IST1: increased sodium tolerance protein 1, ESCRT-III-associated factor
KEGG: Kyoto Encyclopedia of Genes and Genomes
KPNA3: karyopherin subunit alpha 3
MAP2K4: mitogen-activated protein kinase kinase 4
MAPK10: mitogen-activated protein kinase 10
MYCBP2: MYC binding protein 2, E3 ubiquitin protein ligase
NCBI: National Center for Biotechnology Information
NEK2: never in mitosis gene A (NIMA)-related kinase 2
NPC2: Neimann–Pick type C2, intracellular cholesterol transporter
ORF: open reading frame
OTUB1: ovarian tumor domain-containing ubiquitin aldehyde-binding protein 1
PA2G4: proliferation-associated 2G4
PAPOLA: poly(A) polymerase alpha
PARP4: poly(ADP-ribose) polymerase family member 4
PGRMC2: progesterone receptor membrane component 2
PITRM1: pitrilysin metallopeptidase 1
PKD2: polycystin 2, transient receptor potential cation channel
PPI: protein-protein interaction
PPIE: peptidyl-prolyl cis-trans isomerase E
PPM1G: protein phosphatase, Mg2+/Mn2+ dependent 1G
PSMC1: proteasome 26S subunit, ATPase 1
RBBP6: retinoblastoma binding protein 6, ubiquitin ligase
RNF38: ring finger protein 38
RFX5: regulatory factor X5
RGS2: regulator of G-protein signaling 2
ROCK1: Rho-associated coiled-coil containing protein kinase 1
RTN4: reticulon 4
SAP18: sin3A-associated protein 18
SKP1: S-phase kinase-associated protein 1
SNRPA1: small nuclear ribonucleoprotein polypeptide A'
STAG3: stromal antigen 3
T2SS: type II secretion system
T4SS: type IV secretion system
TMED10: transmembrane P24 trafficking protein 10
TMX2: thioredoxin related transmembrane protein 2
TRAPPC8: trafficking protein particle complex 8
TTR: transthyretin
UBE2V2: ubiquitin-conjugating enzyme E2 variant 2
U.S.: Unites States
USP8: ubiquitin specific peptidase 8
USP47: ubiquitin specific peptidase 47
VPS26A: vacuolar protein sorting-associated protein 26A
WDR48: WD repeat domain 48
Y2H: yeast two-hybrid
