# Correction: Mechanisms of action of *Coxiella burnetii* effectors inferred from host-pathogen protein interactions

**DOI:** 10.1371/journal.pone.0196373

**Published:** 2018-04-20

**Authors:** The PLOS ONE Staff

A second version of Table 6 incorrectly replaces Table 7. Please see Table 7 here. The publisher apologizes for the error.

**Table 7:** Unknown secreted effector characterization based on host-protein interaction data.

**Locus**	**Name**	**Pair testing (% identified)**	**Y2H protein interaction data**						
			N_host_	Top locations (%)				Individual interactions	Pathways
CBU0794	coxCC4	22	22{	Nucleus	ER/Golg	Vesicle	Memb	indeterminate	focal adhesion; NF-κB immune-response
				50	27	22	10		
CBU0881	coxCC5	41	71{	Nucleus	Exosome	Centro	ER/Golg	ESCRT; centrosome; transcription	endosomal sorting; focal adhesion; NF-κB immune-response; mitochondrial matrix
				44	24	13	11		
CBU1724	cetCb6	65	50{	Nucleus	ER/Golg	Exosome	Mito	pleiotropic; cholesterol transport	endosomal sorting; focal adhesion; Fc receptor pathway; cell cycle phase transition; mitochondrial matrix
				42	30	28	18		
CBU2078	coxFIC1	67	38{	Nucleus	Exosome	ER/Golg	Memb	endosomal sorting; ubiquitin processing	endosomal sorting; focal adhesion
				50	21	18	18		

Centro, centrosome; ER/Golg, endoplasmic reticulum/Golgi apparatus; ESCRT, *endosomal sorting complexes required for transport*; Memb, plasma membrane; Mito, mitochondria; N_host_, number of host-pathogen protein interactions; Y2H, yeast two-hybrid.
