# Supplementary material for: Mechanisms of action of Coxiella burnetii effectors inferred from host-pathogen protein interactions
Source: PLoS One. 2017 Nov 27;12(11):e0188071. doi: 10.1371/journal.pone.0188071 (PMC5703456; doi:10.1371/journal.pone.0188071)
Supplement: S2 Table — The table provides all pairwise interactions detected in the yeast two-hybrid (Y2H) high-throughput screen from the human libraries and the corresponding murine orthologous interactions as well as the pairwise interactions from the complementary pairwise Y2H testing. (DOCX) [file pone.0188071.s002.docx]

**S2 Table**: ***C. burnetii* protein interaction data.** The table shows all pairwise interactions detected in the yeast two-hybrid (Y2H) high-throughput screen from the human libraries (H) and the corresponding murine orthologous interactions (O). The complementary pairwise Y2H interactions are shown in the last column with the symbols -, Y, N, which indicate not retested, a positive retesting result, and a negative retesting result, respectively.

| ***C. burnetii* gene locus tag** | **Uniprot *C. burnetii* gene ID** | **Human gene ID** | **Uniprot human gene ID** | **High-throughput screening** | **Complementary pairwise screening** |
| --- | --- | --- | --- | --- | --- |
| CBU0041 | Q83FA5 | EIF2AK3 | Q9NZJ5 | H | - |
| CBU0041 | Q83FA5 | MERTK | Q12866 | H | - |
| CBU0041 | Q83FA5 | PLBD1 | Q6P4A8 | H | - |
| CBU0041 | Q83FA5 | AMD1 | P17707 | O | - |
| CBU0041 | Q83FA5 | ASPN | Q9BXN1 | O | - |
| CBU0041 | Q83FA5 | CDK19 | Q9BWU1 | O | - |
| CBU0041 | Q83FA5 | GPM6A | P51674 | O | - |
| CBU0041 | Q83FA5 | HNRNPA3 | P51991 | O | - |
| CBU0041 | Q83FA5 | PCCA | P05165 | O | - |
| CBU0077 | Q83F71 | ACADM | P11310 | H | - |
| CBU0077 | Q83F71 | UBE2B | P63146 | H | - |
| CBU0077 | Q83F71 | WISP1 | O95388 | H | - |
| CBU0077 | Q83F71 | AMICA1 | Q86YT9 | O | - |
| CBU0077 | Q83F71 | CCT2 | P78371 | O | - |
| CBU0077 | Q83F71 | TMED10 | P49755 | O | - |
| CBU0175 | Q820B6 | ANXA5 | P08758 | H | - |
| CBU0175 | Q820B6 | MARCH6 | O60337 | H | - |
| CBU0175 | Q820B6 | CUTC | Q9NTM9 | H | - |
| CBU0175 | Q820B6 | LMAN1 | P49257 | H | - |
| CBU0175 | Q820B6 | OMG | P23515 | H | - |
| CBU0175 | Q820B6 | RBPMS | Q93062 | H | - |
| CBU0175 | Q820B6 | RPS5 | P46782 | H | - |
| CBU0175 | Q820B6 | SNX5 | Q9Y5X3 | H | - |
| CBU0175 | Q820B6 | TRIM6 | Q9C030 | H | - |
| CBU0295 | Q83EM2 | BAZ2B | Q9UIF8 | H | - |
| CBU0295 | Q83EM2 | MRPS12 | O15235 | O | - |
| CBU0295 | Q83EM2 | TDP1 | Q9NUW8 | O | - |
| CBU0295 | Q83EM2 | TRAPPC8 | Q9Y2L5 | O | - |
| CBU0388 | Q83ED7 | RNF2 | Q99496 | H | - |
| CBU0447 | Q83E81 | TES | Q9UGI8 | H | - |
| CBU0781 | Q83DF6 | ABCA1 | O95477 | H | - |
| CBU0781 | Q83DF6 | ATP2B1 | P20020 | H | - |
| CBU0781 | Q83DF6 | ATP6V1D | Q9Y5K8 | H | - |
| CBU0781 | Q83DF6 | ATRNL1 | Q5VV63 | H | - |
| CBU0781 | Q83DF6 | BAZ2B | Q9UIF8 | H | - |
| CBU0781 | Q83DF6 | BBOX1 | O75936 | H | - |
| CBU0781 | Q83DF6 | CA2 | P00918 | H | - |
| CBU0781 | Q83DF6 | CDK14 | O94921 | H | - |
| CBU0781 | Q83DF6 | COMMD10 | Q9Y6G5 | H | - |
| CBU0781 | Q83DF6 | DCLK1 | O15075 | H | - |
| CBU0781 | Q83DF6 | DLAT | P10515 | H | - |
| CBU0781 | Q83DF6 | FCER1G | P30273 | H | - |
| CBU0781 | Q83DF6 | GGH | Q92820 | H | - |
| CBU0781 | Q83DF6 | HNRNPDL | O14979 | H | - |
| CBU0781 | Q83DF6 | KPNA3 | O00505 | H | - |
| CBU0781 | Q83DF6 | LGMN | Q99538 | H | - |
| CBU0781 | Q83DF6 | LTBP1 | Q14766 | H | - |
| CBU0781 | Q83DF6 | OCIAD1 | Q9NX40 | H | - |
| CBU0781 | Q83DF6 | PAH | P00439 | H | - |
| CBU0781 | Q83DF6 | PARP4 | Q9UKK3 | H | - |
| CBU0781 | Q83DF6 | PEX5L | Q8IYB4 | H | - |
| CBU0781 | Q83DF6 | PGRMC2 | O15173 | H | - |
| CBU0781 | Q83DF6 | RFX5 | P48382 | H | - |
| CBU0781 | Q83DF6 | SLC5A12 | Q1EHB4 | H | - |
| CBU0781 | Q83DF6 | STMN2 | Q93045 | H | - |
| CBU0781 | Q83DF6 | TMEM183A | Q8IXX5 | H | - |
| CBU0781 | Q83DF6 | VKORC1L1 | Q8N0U8 | H | - |
| CBU0781 | Q83DF6 | ZBED4 | O75132 | H | - |
| CBU0781 | Q83DF6 | ZC3HC | Q8WU90 | H | - |
| CBU0781 | Q83DF6 | ZC3H8 | Q8N5P1 | H | - |
| CBU0781 | Q83DF6 | ZDHHC6 | Q9H6R6 | H | - |
| CBU0781 | Q83DF6 | ZNF302 | Q9NR11 | H | - |
| CBU0781 | Q83DF6 | ZNF623 | O75123 | H | - |
| CBU0781 | Q83DF6 | ASPN | Q9BXN1 | O | - |
| CBU0781 | Q83DF6 | ECH1 | Q13011 | O | - |
| CBU0781 | Q83DF6 | GABBR2 | O75899 | O | - |
| CBU0781 | Q83DF6 | SCEL | O95171 | O | - |
| CBU0781 | Q83DF6 | STAG3 | Q9UJ98 | O | - |
| CBU0781 | Q83DF6 | VIM | P08670 | O | - |
| CBU0781 | Q83DF6 | ZNF706 | Q9Y5V0 | O | - |
| CBU0794 | Q83DE4 | ANGPT1 | Q15389 | H | - |
| CBU0794 | Q83DE4 | CALCOCO2 | Q13137 | H | N |
| CBU0794 | Q83DE4 | CBY1 | Q9Y3M2 | H | - |
| CBU0794 | Q83DE4 | FAM73A | Q8NAN2 | H | - |
| CBU0794 | Q83DE4 | FMNL2 | Q96PY5 | H | N |
| CBU0794 | Q83DE4 | LIN7C | Q9NUP9 | H | - |
| CBU0794 | Q83DE4 | MECOM | Q03112 | H | N |
| CBU0794 | Q83DE4 | NRP1 | O14786 | H | - |
| CBU0794 | Q83DE4 | OSBPL9 | Q96SU4 | H | - |
| CBU0794 | Q83DE4 | PPP2CB | P62714 | H | N |
| CBU0794 | Q83DE4 | PSMC1 | P62191 | H | N |
| CBU0794 | Q83DE4 | RAI14 | Q9P0K7 | H | - |
| CBU0794 | Q83DE4 | SPATA22 | Q8NHS9 | H | - |
| CBU0794 | Q83DE4 | SYT4 | Q9H2B2 | H | - |
| CBU0794 | Q83DE4 | ZNF514 | Q96K75 | H | N |
| CBU0794 | Q83DE4 | ZNF583 | Q96ND8 | H | Y |
| CBU0794 | Q83DE4 | ZNF638 | Q14966 | H | - |
| CBU0794 | Q83DE4 | BBS5 | Q8N3I7 | O | Y |
| CBU0794 | Q83DE4 | CAPN2 | P17655 | O | - |
| CBU0794 | Q83DE4 | RDM1 | Q8NG50 | O | N |
| CBU0794 | Q83DE4 | SCG3 | Q8WXD2 | O | - |
| CBU0794 | Q83DE4 | SEC24B | O95487 | O | - |
| CBU0881 | Q83D60 | ALDH6A1 | Q02252 | H | N |
| CBU0881 | Q83D60 | ANKRD7 | Q92527 | H | - |
| CBU0881 | Q83D60 | APH1B | Q8WW43 | H | - |
| CBU0881 | Q83D60 | ARID2 | Q68CP9 | H | Y |
| CBU0881 | Q83D60 | C22orf23 | Q9BZE7 | H | - |
| CBU0881 | Q83D60 | C8orf48 | Q96LL4 | H | - |
| CBU0881 | Q83D60 | CAPN7 | Q9Y6W3 | H | - |
| CBU0881 | Q83D60 | CCDC181 | Q5TID7 | H | - |
| CBU0881 | Q83D60 | CCT2 | P78371 | H | N |
| CBU0881 | Q83D60 | CLIC1 | O00299 | H | - |
| CBU0881 | Q83D60 | COPS4 | Q9BT78 | H | - |
| CBU0881 | Q83D60 | DCAF6 | Q58WW2 | H | Y |
| CBU0881 | Q83D60 | DCTN6 | O00399 | H | Y |
| CBU0881 | Q83D60 | EFHC1 | Q5JVL4 | H | Y |
| CBU0881 | Q83D60 | ENKUR | Q8TC29 | H | - |
| CBU0881 | Q83D60 | F5 | P12259 | H | - |
| CBU0881 | Q83D60 | FAM45A | Q8TCE6 | H | - |
| CBU0881 | Q83D60 | FAM60A | Q9NP50 | H | Y |
| CBU0881 | Q83D60 | FRY | Q5TBA9 | H | - |
| CBU0881 | Q83D60 | FUCA1 | P04066 | H | N |
| CBU0881 | Q83D60 | GDAP2 | Q9NXN4 | H | Y |
| CBU0881 | Q83D60 | GDI2 | P50395 | H | N |
| CBU0881 | Q83D60 | HYLS1 | Q96M11 | H | - |
| CBU0881 | Q83D60 | IARS2 | Q9NSE4 | H | Y |
| CBU0881 | Q83D60 | IFT81 | Q8WYA0 | H | N |
| CBU0881 | Q83D60 | IK | Q13123 | H | - |
| CBU0881 | Q83D60 | IST1 | P53990 | H | - |
| CBU0881 | Q83D60 | LRRC6 | Q86X45 | H | - |
| CBU0881 | Q83D60 | MRPL48 | Q96GC5 | H | - |
| CBU0881 | Q83D60 | MTIF2 | Q6P1N2 | H | N |
| CBU0881 | Q83D60 | MYC | P01106 | H | - |
| CBU0881 | Q83D60 | NEK2 | P51955 | H | Y |
| CBU0881 | Q83D60 | NFIA | Q12857 | H | - |
| CBU0881 | Q83D60 | PGAM1 | P18669 | H | - |
| CBU0881 | Q83D60 | PITRM1 | Q5JRX3 | H | Y |
| CBU0881 | Q83D60 | PLOD2 | O00469 | H | - |
| CBU0881 | Q83D60 | PMS1 | P54277 | H | N |
| CBU0881 | Q83D60 | PPA1 | Q15181 | H | - |
| CBU0881 | Q83D60 | PPIE | Q9UNP9 | H | Y |
| CBU0881 | Q83D60 | PSMB1 | P20618 | H | N |
| CBU0881 | Q83D60 | PUM1 | Q14671 | H | - |
| CBU0881 | Q83D60 | RGS7 | P49802 | H | Y |
| CBU0881 | Q83D60 | RPS8 | P62241 | H | N |
| CBU0881 | Q83D60 | SH3YL1 | Q96HL8 | H | N |
| CBU0881 | Q83D60 | SLC7A13 | Q8TCU3 | H | N |
| CBU0881 | Q83D60 | SPATA22 | Q8NHS9 | H | - |
| CBU0881 | Q83D60 | SYCP1 | Q15431 | H | N |
| CBU0881 | Q83D60 | THBS3 | P49746 | H | N |
| CBU0881 | Q83D60 | TOPBP1 | Q92547 | H | N |
| CBU0881 | Q83D60 | TRIM22 | Q8IYM9 | H | - |
| CBU0881 | Q83D60 | UBAP2L | Q14157 | H | - |
| CBU0881 | Q83D60 | USP8 | P40818 | H | N |
| CBU0881 | Q83D60 | ZBTB4 | Q9P1Z0 | H | N |
| CBU0881 | Q83D60 | ZIM2 | O18067 | H | - |
| CBU0881 | Q83D60 | ZMYM2 | Q9UBW7 | H | Y |
| CBU0881 | Q83D60 | ZNF177 | Q13360 | H | - |
| CBU0881 | Q83D60 | ZNF350 | Q9GZX5 | H | N |
| CBU0881 | Q83D60 | ZNF438 | Q7Z4V0 | H | N |
| CBU0881 | Q83D60 | ASRGL1 | Q7L266 | O | Y |
| CBU0881 | Q83D60 | ATP6V1E2 | Q96A05 | O | - |
| CBU0881 | Q83D60 | BBS5 | Q8N3I7 | O | Y |
| CBU0881 | Q83D60 | CYB5R4 | Q7L1T6 | O | N |
| CBU0881 | Q83D60 | EPHX2 | P34913 | O | - |
| CBU0881 | Q83D60 | EPRS | P07814 | O | - |
| CBU0881 | Q83D60 | FAM227B | Q96M60 | O | - |
| CBU0881 | Q83D60 | FASTKD5 | Q7L8L6 | O | - |
| CBU0881 | Q83D60 | G6PD | P11413 | O | - |
| CBU0881 | Q83D60 | KLHL13 | Q9P2N7 | O | - |
| CBU0881 | Q83D60 | SCAF4 | O95104 | O | - |
| CBU0881 | Q83D60 | SPARCL1 | Q14515 | O | N |
| CBU0881 | Q83D60 | UMOD | P07911 | O | - |
| CBU0881 | Q83D60 | MTIF2 | Q6P1N2 | H | - |
| CBU0885 | Q83D57 | CENPC | Q03188 | H | - |
| CBU0885 | Q83D57 | ZNF595 | Q8IYB9 | H | - |
| CBU0937 | Q83D09 | ADH1B | P00325 | H | - |
| CBU0937 | Q83D09 | C7orf60 | Q1RMZ1 | H | - |
| CBU0937 | Q83D09 | CAST | P20810 | H | - |
| CBU0937 | Q83D09 | EDNRB | P24530 | H | - |
| CBU0937 | Q83D09 | EIF1B | O60739 | H | - |
| CBU0937 | Q83D09 | FAM214A | Q32MH5 | h | - |
| CBU0937 | Q83D09 | FKBP7 | Q9Y680 | H | - |
| CBU0937 | Q83D09 | GPR137C | Q8N3F9 | H | - |
| CBU0937 | Q83D09 | HOOK1 | Q9UJC3 | H | - |
| CBU0937 | Q83D09 | LRRC49 | Q8IUZ0 | H | - |
| CBU0937 | Q83D09 | MAN1A1 | P33908 | H | - |
| CBU0937 | Q83D09 | MAP2K4 | P45985 | H | - |
| CBU0937 | Q83D09 | MRPL27 | Q9P0M9 | H | - |
| CBU0937 | Q83D09 | NCKAP1 | Q9Y2A7 | H | - |
| CBU0937 | Q83D09 | PA2G4 | Q9UQ80 | H | - |
| CBU0937 | Q83D09 | PAPOLA | P51003 | H | - |
| CBU0937 | Q83D09 | PDZK1 | Q5T2W1 | H | - |
| CBU0937 | Q83D09 | PPM1G | O15355 | H | - |
| CBU0937 | Q83D09 | PTER | Q96BW5 | H | - |
| CBU0937 | Q83D09 | RGS2 | P41220 | H | - |
| CBU0937 | Q83D09 | RPRD1B | Q9NQG5 | H | - |
| CBU0937 | Q83D09 | RPUSD4 | Q96CM3 | H | - |
| CBU0937 | Q83D09 | SCN3B | Q9NY72 | H | - |
| CBU0937 | Q83D09 | SCP2 | P22307 | H | - |
| CBU0937 | Q83D09 | SLC22A24 | Q8N4F4 | H | - |
| CBU0937 | Q83D09 | ZNFX1 | Q9P2E3 | H | - |
| CBU1314 | Q83C21 | PSMA3 | P25788 | H | - |
| CBU1314 | Q83C21 | PSMC1 | P62191 | H | - |
| CBU1314 | Q83C21 | SCEL | O95171 | H | - |
| CBU1314 | Q83C21 | FLNA | P21333 | O | - |
| CBU1379a | B5QSC8 | BAZ2B | Q9UIF8 | H | - |
| CBU1379a | B5QSC8 | CSDE1 | O75534 | H | - |
| CBU1379a | B5QSC8 | DNAJB11 | Q9UBS4 | H | - |
| CBU1379a | B5QSC8 | FBN1 | P35555 | H | - |
| CBU1379a | B5QSC8 | MAGED1 | Q9Y5V3 | H | - |
| CBU1379a | B5QSC8 | METTL7A | Q9H8H3 | H | - |
| CBU1379a | B5QSC8 | ZIC2 | O95409 | h | - |
| CBU1457 | Q83BP6 | COL2A1 | P02458 | H | - |
| CBU1457 | Q83BP6 | CPD | O75976 | H | - |
| CBU1457 | Q83BP6 | DHX9 | Q08211 | H | - |
| CBU1457 | Q83BP6 | ETFA | P13804 | H | - |
| CBU1457 | Q83BP6 | FAM177A1 | Q8N128 | H | - |
| CBU1457 | Q83BP6 | ITGAV | P06756 | H | - |
| CBU1457 | Q83BP6 | MGAM | O43451 | H | - |
| CBU1457 | Q83BP6 | SH3BGRL | O75368 | H | - |
| CBU1457 | Q83BP6 | TMEM106B | Q9NUM4 | H | - |
| CBU1457 | Q83BP6 | ABI1 | Q8IZP0 | O | - |
| CBU1457 | Q83BP6 | ASPA | P45381 | O | - |
| CBU1457 | Q83BP6 | ASRGL1 | Q7L266 | O | - |
| CBU1457 | Q83BP6 | CLK1 | P49759 | O | - |
| CBU1457 | Q83BP6 | CUL1 | Q13616 | O | - |
| CBU1457 | Q83BP6 | DCTN6 | O00399 | O | - |
| CBU1457 | Q83BP6 | FKBP1A | P62942 | O | - |
| CBU1457 | Q83BP6 | FRYL | O94915 | O | - |
| CBU1457 | Q83BP6 | GPHN | Q9NQX3 | O | - |
| CBU1457 | Q83BP6 | HSPA5 | P11021 | O | - |
| CBU1457 | Q83BP6 | NHEJ1 | Q9H9Q4 | O | - |
| CBU1457 | Q83BP6 | NUDCD2 | Q8WVJ2 | O | - |
| CBU1457 | Q83BP6 | NXT1 | Q9UKK6 | O | - |
| CBU1457 | Q83BP6 | PLS1 | Q14651 | O | - |
| CBU1457 | Q83BP6 | PPP1R42 | Q7Z4L9 | O | - |
| CBU1457 | Q83BP6 | RARB | P10826 | O | - |
| CBU1457 | Q83BP6 | RNF19A | Q9NV58 | O | - |
| CBU1457 | Q83BP6 | STK39 | Q9UEW8 | O | - |
| CBU1457 | Q83BP6 | SYNJ1 | Q05CZ1 | O | - |
| CBU1457 | Q83BP6 | TOLLIP | Q9H0E2 | O | - |
| CBU1457 | Q83BP6 | TRAPPC3 | O43617 | O | - |
| CBU1457 | Q83BP6 | VCL | P18206 | O | - |
| CBU1457 | Q83BP6 | WDR61 | Q9GZS3 | O | - |
| CBU1457 | Q83BP6 | ZC3HC1 | Q86WB0 | O | - |
| CBU1460 | Q83BP3 | TFCP2L1 | Q9NZI6 | O | - |
| CBU1460 | Q83BP3 | C5Orf63 | A6NC05 | O | - |
| CBU1460 | Q83BP3 | CHRAC1 | Q9NRG0 | O | - |
| CBU1460 | Q83BP3 | LPAR1 | Q6GPG7 | O | - |
| CBU1460 | Q83BP3 | SMARCA2 | P51531 | O | - |
| CBU1524 | NA | CFAP97 | Q9P2B7 | H | - |
| CBU1524 | NA | CMTM6 | Q9NX76 | H | - |
| CBU1524 | NA | DHX15 | O43143 | H | - |
| CBU1524 | NA | ENY2 | Q9NPA8 | H | - |
| CBU1524 | NA | EPCAM | P16422 | H | - |
| CBU1524 | NA | ERLIN2 | O94905 | H | - |
| CBU1524 | NA | ETS2 | P15036 | H | - |
| CBU1524 | NA | ETV1 | P50549 | H | - |
| CBU1524 | NA | FBL | P22087 | H | - |
| CBU1524 | NA | FYTTD1 | Q96QD9 | H | - |
| CBU1524 | NA | H2AFY | O75367 | H | - |
| CBU1524 | NA | HNRNPM | P52272 | H | - |
| CBU1524 | NA | HRSP12 | P52758 | H | - |
| CBU1524 | NA | LMAN1 | P49257 | H | - |
| CBU1524 | NA | MGAM | O43451 | H | - |
| CBU1524 | NA | PKD2 | Q13563 | H | - |
| CBU1524 | NA | PLP1 | P60201 | H | - |
| CBU1524 | NA | PSMA6 | P60900 | H | - |
| CBU1524 | NA | PSMA7 | O14818 | H | - |
| CBU1524 | NA | RNF38 | Q9H0F5 | H | - |
| CBU1524 | NA | RTN4 | Q9NQC3 | H | - |
| CBU1524 | NA | SAP18 | O00422 | H | - |
| CBU1524 | NA | SLC35F1 | Q5T1Q4 | H | - |
| CBU1524 | NA | SLC7A13 | Q8TCU3 | H | - |
| CBU1524 | NA | SNRPA1 | P09661 | H | - |
| CBU1524 | NA | SRP9 | P49458 | H | - |
| CBU1524 | NA | TARBP1 | Q13395 | H | - |
| CBU1524 | NA | TCF4 | P15884 | H | - |
| CBU1524 | NA | TXN | P10599 | H | - |
| CBU1524 | NA | TXNL1 | O43396 | H | - |
| CBU1524 | NA | UNC13B | I6L9J0 | H | - |
| CBU1524 | NA | USP8 | P40818 | H | - |
| CBU1524 | NA | ZBTB21 | Q9ULJ3 | H | - |
| CBU1524 | NA | ZMYM2 | Q9UBW7 | H | - |
| CBU1524 | NA | ZNF226 | Q9NYT6 | H | - |
| CBU1543 | Q83BG5 | SNN | O75324 | O | - |
| CBU1556 | Q83BF4 | PSMA7 | O14818 | H | - |
| CBU1556 | Q83BF4 | CLK4 | Q9HAZ1 | O | - |
| CBU1556 | Q83BF4 | GPAM | Q9HCL2 | O | - |
| CBU1556 | Q83BF4 | RBL2 | Q08999 | O | - |
| CBU1724 | Q83B01 | AK3 | Q9UIJ7 | H | Y |
| CBU1724 | Q83B01 | APOA1BP | Q8NCW5 | H | Y |
| CBU1724 | Q83B01 | CCT6A | P40227 | H | N |
| CBU1724 | Q83B01 | CDK19 | Q9BWU1 | H | - |
| CBU1724 | Q83B01 | CUL1 | Q13616 | H | N |
| CBU1724 | Q83B01 | DHX8 | Q14562 | H | Y |
| CBU1724 | Q83B01 | DKK3 | Q9UBP4 | H | Y |
| CBU1724 | Q83B01 | DPY30 | Q9C005 | H | - |
| CBU1724 | Q83B01 | FEZ2 | Q9UHY8 | H | - |
| CBU1724 | Q83B01 | HSPA14 | Q0VDF9 | H | Y |
| CBU1724 | Q83B01 | MAPK10 | P53779 | H | Y |
| CBU1724 | Q83B01 | NIFK | Q9BYG3 | H | - |
| CBU1724 | Q83B01 | RAB18 | Q9NP72 | H | - |
| CBU1724 | Q83B01 | SLC2A1 | P11166 | H | - |
| CBU1724 | Q83B01 | TRAPPC13 | A5PLN9 | H | - |
| CBU1724 | Q83B01 | TRAPPC8 | Q9Y2L5 | H | N |
| CBU1724 | Q83B01 | TXNL1 | O43396 | H | Y |
| CBU1724 | Q83B01 | ACO1 | P21399 | O | - |
| CBU1724 | Q83B01 | AIDA | Q96BJ3 | O | - |
| CBU1724 | Q83B01 | ANLN | Q9NQW6 | O | Y |
| CBU1724 | Q83B01 | AP3B1 | O00203 | O | - |
| CBU1724 | Q83B01 | ARCN1 | P48444 | O | - |
| CBU1724 | Q83B01 | ASCC3 | Q8N3C0 | O | - |
| CBU1724 | Q83B01 | ASPA | P45381 | O | - |
| CBU1724 | Q83B01 | ASS1 | P00966 | O | - |
| CBU1724 | Q83B01 | ATXN10 | Q9UBB4 | O | - |
| CBU1724 | Q83B01 | BBS7 | Q8IWZ6 | O | - |
| CBU1724 | Q83B01 | COPG2 | Q9UBF2 | O | - |
| CBU1724 | Q83B01 | CYB5R4 | Q7L1T6 | O | Y |
| CBU1724 | Q83B01 | DNAJA1 | P31689 | O | Y |
| CBU1724 | Q83B01 | EIF3A | Q14152 | O | - |
| CBU1724 | Q83B01 | FLRT2 | O43155 | O | - |
| CBU1724 | Q83B01 | INPP1 | P49441 | O | - |
| CBU1724 | Q83B01 | LHCGR | P22888 | O | - |
| CBU1724 | Q83B01 | MCCC1 | Q96RQ3 | O | N |
| CBU1724 | Q83B01 | MYCBP2 | O75592 | O | - |
| CBU1724 | Q83B01 | NEMF | O60524 | O | N |
| CBU1724 | Q83B01 | NPC2 | P61916 | O | Y |
| CBU1724 | Q83B01 | OTUB1 | Q96FW1 | O | Y |
| CBU1724 | Q83B01 | OXCT2 | Q9BYC2 | O | N |
| CBU1724 | Q83B01 | PPP1R42 | Q7Z4L9 | O | - |
| CBU1724 | Q83B01 | PRMT9 | Q6P2P2 | O | - |
| CBU1724 | Q83B01 | PRMT3 | O60678 | O | - |
| CBU1724 | Q83B01 | QARS | P47897 | O | - |
| CBU1724 | Q83B01 | RYR3 | Q15413 | O | - |
| CBU1724 | Q83B01 | TMIGD1 | Q6UXZ0 | O | - |
| CBU1724 | Q83B01 | TTR | P02766 | O | - |
| CBU1724 | Q83B01 | UBE2V2 | Q15819 | O | N |
| CBU1724 | Q83B01 | WDR61 | Q9GZS3 | O | Y |
| CBU1724 | Q83B01 | YAE1D1 | Q9NRH1 | O | - |
| CBU1751 | Q83AX6 | GLUD1 | P00367 | O | - |
| CBU1751 | Q83AX6 | SPARCL1 | Q14515 | O | - |
| CBU1769 | Q83AV9 | CPSF3 | Q9UKF6 | H | - |
| CBU1769 | Q83AV9 | HNRNPLL | Q8WVV9 | H | - |
| CBU1769 | Q83AV9 | MLLT3 | P42568 | H | - |
| CBU1769 | Q83AV9 | STX8 | Q9UNK0 | H | - |
| CBU1769 | Q83AV9 | YME1L1 | Q96TA2 | H | - |
| CBU1825 | Q83AQ5 | WNK1 | Q9H4A3 | O | - |
| CBU1825 | Q83AQ5 | ETFA | P13804 | O | - |
| CBU1825 | Q83AQ5 | COMMD8 | Q9NX08 | O | - |
| CBU1825 | Q83AQ5 | YME1L1 | Q96TA2 | O | - |
| CBU1825 | Q83AQ5 | HMOX2 | P30519 | O | - |
| CBU2056 | Q83A54 | H3AFY | O75367 | O | - |
| CBU2056 | Q83A54 | SPARCL1 | Q14515 | O | - |
| CBU2056 | Q83A54 | GGNBP2 | Q9H3C7 | O | - |
| CBU2078 | Q83A33 | C11orf74 | Q86VG3 | H | - |
| CBU2078 | Q83A33 | DCTN6 | O00399 | H | Y |
| CBU2078 | Q83A33 | EIF4A2 | Q14240 | H | - |
| CBU2078 | Q83A33 | HSPA5 | P11021 | H | N |
| CBU2078 | Q83A33 | ITGA2 | P17301 | H | - |
| CBU2078 | Q83A33 | MBNL1 | Q9NR56 | H | - |
| CBU2078 | Q83A33 | MGAM | O43451 | H | - |
| CBU2078 | Q83A33 | MTIF2 | Q6P1N2 | H | - |
| CBU2078 | Q83A33 | RNF38 | Q9H0F5 | H | Y |
| CBU2078 | Q83A33 | RSL24D1 | Q9UHA3 | H | - |
| CBU2078 | Q83A33 | SAMD9L | Q8IVG5 | H | - |
| CBU2078 | Q83A33 | SLC35A5 | Q9BS91 | H | - |
| CBU2078 | Q83A33 | TRAPPC8 | Q9Y2L5 | H | N |
| CBU2078 | Q83A33 | USP8 | P40818 | H | N |
| CBU2078 | Q83A33 | WDR48 | Q8TAF3 | H | Y |
| CBU2078 | Q83A33 | ARCN1 | P48444 | O | - |
| CBU2078 | Q83A33 | ATXN10 | Q9UBB4 | O | - |
| CBU2078 | Q83A33 | BCLAF1 | Q9NYF8 | O | - |
| CBU2078 | Q83A33 | CEP97 | Q8IW35 | O | - |
| CBU2078 | Q83A33 | CYB5R4 | Q7L1T6 | O | Y |
| CBU2078 | Q83A33 | DNAJA2 | O60884 | O | - |
| CBU2078 | Q83A33 | DNAJB4 | Q9UDY4 | O | - |
| CBU2078 | Q83A33 | FARSB | Q9NSD9 | O | - |
| CBU2078 | Q83A33 | H2AFY | O75367 | O | - |
| CBU2078 | Q83A33 | KATNBL1 | Q9H079 | O | - |
| CBU2078 | Q83A33 | MTTP | P55157 | O | - |
| CBU2078 | Q83A33 | NLRP14 | Q86W24 | O | - |
| CBU2078 | Q83A33 | NPC2 | P61916 | O | Y |
| CBU2078 | Q83A33 | OXCT2 | Q9BYC2 | O | - |
| CBU2078 | Q83A33 | PDZD8 | Q8NEN9 | O | - |
| CBU2078 | Q83A33 | PSMA4 | P25789 | O | - |
| CBU2078 | Q83A33 | RBBP6 | Q7Z6E9 | O | Y |
| CBU2078 | Q83A33 | TCEA1 | P23193 | O | - |
| CBU2078 | Q83A33 | TMEM56 | Q96MV1 | O | - |
| CBU2078 | Q83A33 | UCHL3 | P15374 | O | N |
| CBU2078 | Q83A33 | USP47 | Q96K76 | O | Y |
| CBU2078 | Q83A33 | WDR61 | Q9GZS3 | O | Y |
| CBU2078 | Q83A33 | ZFP36L2 | P47974 | O | - |
| CBUA0014 | Q83A11 | ACSM2A | Q08AH3 | H | - |
| CBUA0014 | Q83A11 | ARHGAP5 | Q13017 | H | - |
| CBUA0014 | Q83A11 | ARHGDIB | P52566 | H | N |
| CBUA0014 | Q83A11 | CMPK1 | P30085 | H | - |
| CBUA0014 | Q83A11 | DNAJB11 | Q9UBS4 | H | N |
| CBUA0014 | Q83A11 | FBN1 | P35555 | H | - |
| CBUA0014 | Q83A11 | HSPA14 | Q0VDF9 | H | Y |
| CBUA0014 | Q83A11 | KRAS | P01116 | H | - |
| CBUA0014 | Q83A11 | LPL | P06858 | H | N |
| CBUA0014 | Q83A11 | LRRC6 | Q86X45 | H | - |
| CBUA0014 | Q83A11 | LSM5 | Q9Y4Y9 | H | - |
| CBUA0014 | Q83A11 | MPDZ | O75970 | H | Y |
| CBUA0014 | Q83A11 | MTDH | Q86UE4 | H | N |
| CBUA0014 | Q83A11 | PSMA7 | O14818 | H | N |
| CBUA0014 | Q83A11 | QKI | Q96PU8 | H | - |
| CBUA0014 | Q83A11 | SCAF4 | O95104 | H | - |
| CBUA0014 | Q83A11 | SEH1L | Q96EE3 | H | - |
| CBUA0014 | Q83A11 | SLC7A13 | Q8TCU3 | H | N |
| CBUA0014 | Q83A11 | TMED5 | Q9Y3A6 | H | N |
| CBUA0014 | Q83A11 | TMX2 | Q9Y320 | H | Y |
| CBUA0014 | Q83A11 | TRAPPC8 | Q9Y2L5 | H | N |
| CBUA0014 | Q83A11 | TRIM2 | Q9C040 | H | N |
| CBUA0014 | Q83A11 | VPS26A | O75436 | H | Y |
| CBUA0014 | Q83A11 | WDR48 | Q8TAF3 | H | Y |
| CBUA0014 | Q83A11 | WWC1 | Q8IX03 | H | - |
| CBUA0014 | Q83A11 | ZNF146 | Q15072 | H | - |
| CBUA0014 | Q83A11 | ATXN10 | Q9UBB4 | O | - |
| CBUA0014 | Q83A11 | GANC | Q8TET4 | O | - |
| CBUA0014 | Q83A11 | GK | P32189 | O | - |
| CBUA0014 | Q83A11 | HNRNPH3 | P31942 | O | - |
| CBUA0014 | Q83A11 | NPM1 | P06748 | O | N |
| CBUA0014 | Q83A11 | SKP1 | P63208 | O | Y |
